# Immune checkpoint of B7-H3 in cancer: from immunology to clinical immunotherapy

**DOI:** 10.1186/s13045-022-01364-7

**Published:** 2022-10-25

**Authors:** Binghao Zhao, Huanzhang Li, Yu Xia, Yaning Wang, Yuekun Wang, Yixin Shi, Hao Xing, Tian Qu, Yu Wang, Wenbin Ma

**Affiliations:** 1 Department of Neurosurgery, Peking Union Medical College Hospital, Chinese Academy of Medical Sciences and Peking Union Medical College, Beijing, 100730 People’s Republic of China; 2 State Key Laboratory of Complex Severe and Rare Diseases, Peking Union Medical College Hospital, Chinese Academy of Medical Science and Peking Union Medical College, Beijing, People’s Republic of China

**Keywords:** B7-H3, Tumor microenvironment, Cancer immune checkpoints, Cancer immunotherapy, Biomarker

## Abstract

Immunotherapy for cancer is a rapidly developing treatment that modifies the immune system and enhances the antitumor immune response. B7-H3 (CD276), a member of the B7 family that plays an immunoregulatory role in the T cell response, has been highlighted as a novel potential target for cancer immunotherapy. B7-H3 has been shown to play an inhibitory role in T cell activation and proliferation, participate in tumor immune evasion and influence both the immune response and tumor behavior through different signaling pathways. B7-H3 expression has been found to be aberrantly upregulated in many different cancer types, and an association between B7-H3 expression and poor prognosis has been established. Immunotherapy targeting B7-H3 through different approaches has been developing rapidly, and many ongoing clinical trials are exploring the safety and efficacy profiles of these therapies in cancer. In this review, we summarize the emerging research on the function and underlying pathways of B7-H3, the expression and roles of B7-H3 in different cancer types, and the advances in B7-H3-targeted therapy. Considering different tumor microenvironment characteristics and results from preclinical models to clinical practice, the research indicates that B7-H3 is a promising target for future immunotherapy, which might eventually contribute to an improvement in cancer immunotherapy that will benefit patients.

## Importance of this study

1. We comprehensively reviewed the literature concerning B7-H3, from the biological features of B7-H3 to the roles of B7-H3 in the TME and malignant tumor behaviors, and discuss newly emerging evidence.
2. We interpreted the relationship between B7-H3 and multiple TME characteristics and summarized the signaling pathways involved in tumorigenesis and the therapeutic approaches developed on the basis of previous studies, fueling the application of targeting B7-H3 from bench to bedside.
3. We comprehensively reviewed the distribution, expression and function of potential receptors that were recently discovered using high-throughput methods.
4. We reviewed the research progress on B7-H3 in different solid cancer types, pointing out the research lacking in the field and conflicting evidence that needs further verification.
5. We summarized rapidly developing clinical trials targeting B7-H3 and other clinical applications of B7-H3.

## Introduction

**Fig. 1:**
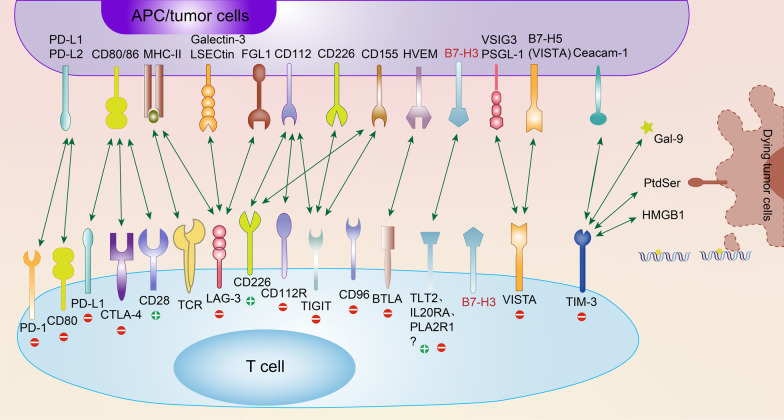
Current immune checkpoint receptors and their respective ligands. Many immune checkpoints expressed on the surface of T cells, such as PD-1, CTLA-4, LAG-3, TIGIT, VISTA, and TIM-3, bind to their respective ligands on APCs and/or tumor cells, eliciting positive and/or negative activity in the T cell response. TIM-3 also participates in associated signaling through PtdSer, HMGB-1 and Gal-9 in dying tumor cells. Notably, checkpoints such as PD-L1, CD80, CD226, and VISTA (B7-H5) are expressed on both T cells and APC/tumor cells. B7-H3 is also expressed on the surface of both T cells and APC/tumor cells, but its receptors have not been clearly elucidated, which has engendered great enthusiasm in cancer immunology investigators. In this article, we identify TLT-2, IL20RA, and PLA2R1 as three potential receptors for B7-H3. “+” in green indicates the immunostimulatory (positive) signal, and “−” in red indicates the immunosuppressive (negative) signal. PtdSer, phosphatidylserine; HMGB-1, high-mobility group protein B1; Gal-9, galectin-9

Immune checkpoints are a group of cell surface proteins that provide either activating or inhibitory signals to control the initiation, duration and magnitude of the immune response [1]. These inhibitory immune checkpoints usually function as a brake to prevent T cell death, reduce damage in healthy tissue and maintain self-tolerance and homeostasis [2], while in cancer, these checkpoints contribute to the ability of cancer cells to evade immune destruction, which is often cited as a “hallmark of cancer,” providing therapeutic targets for rapidly developing immune-oncological drugs, such as immune-activating PD-1 monoclonal antibodies [3]. The efficacy of antibodies targeting immune checkpoints has been verified in several clinical trials, mostly targeting CTLA-4 and PD-1/PD-L1 [4–6]. The great potential of these immune checkpoint inhibitor (ICI) therapies in both preclinical models and clinical trials has greatly sparked scientific interest and made immune checkpoint inhibitors a rapidly developing field, with ICIs alone or in combination being evaluated in 5683 active clinical trials in 2021 [7]. Figure 1 summarizes the commonly used checkpoints of potential translational value. Nevertheless, many patients are still unresponsive to the available ICI therapies, indicating a need to explore the underlying mechanism and other potential targets [8].

B7 family members have been identified as a group of immune regulatory ligands that modulate T lymphocyte activation and differentiation and exhibit a marked interaction with the CD28 superfamily. They are expressed extensively in adaptive and innate immune cells as well as in various cancer tissues, contributing to cancer immune evasion capacity [9]. In addition to the well-studied B7-H1 (PD-L1), the B7 family consists of ten members in total: B7-1 (CD80), B7-2 (CD86), B7-DC (PD-L2), B7-H2 (CD275), B7-H3 (CD276), B7-H4, B7-H5, B7-H6, and B7-H7 (HHLA2) [10]. As a member of the B7 family, B7-H3 has gained great attention in the last decade since its discovery in 2001 [11]. B7-H3 has shown a seemingly contradictory role in T cell activation, while the nature of the B7-H3 receptor has not been clearly elucidated. It has been demonstrated that B7-H3 contributes to tumorigenesis, metastasis and malignant behaviors through various mechanisms, and an association between B7-H3 expression and poor prognosis has been established. To further extend our knowledge of cancer immunotherapy and fuel clinical research targeting B7-H3, an up-to-date and comprehensive review is needed as the literature regarding B7-H3 is rapidly accumulating. Herein, in this review, we summarize the latest research on the function and underlying pathways of B7-H3, the expression and roles of B7-H3 in different cancer types, and the advances in B7-H3 immunotherapy in clinical trials.

## Structure of B7-H3

The human B7-H3 gene locates in 15q24.1 and has 12 exons encoding 316 amino acids, it is a type 1 transmembrane glycoprotein with two isoforms: 2IgB7-H3 (B7-H3 VC) and 4IgB7-H3 (B7-H3b or B7-H3 VCVC) [11, 12]. The 2IgB7-H3 structure comprises single extracellular V- and C-like Ig domains, a transmembrane region and a 45-aa cytoplasmic tail, which was described in an early study using nucleic acid sequence analysis in a human dendritic cell (DC)-derived cDNA library [11]. The presence of the 4IgB7-H3 isoform with two identical pairs of IgV-like and IgC-like domains was later verified in humans [13]. In humans, 4IgB7-H3 is the major isoform expressed on immunocytes as well as on malignant cells [14]. The murine B7-H3 gene locates in chromosome 9, it has a structure similar to that of human 2IgB7-H3, with 93% amino acid similarity [13]. The predicted molecular weight of 2IgB7-H3 is ~ 70 kDa based on the amino acid sequence, while B7-H3 was detected as an ~ 110 kDa glycoprotein via western blotting in human breast cancer samples [15]. The crystal structure of murine B7-H3 has been reported, suggesting that the FG loop of the IgV domain plays a critical role in its inhibitory function [16]. In addition to the transmembrane form, soluble B7-H3 (sB7-H3) has been detected in normal human serum [17]. sB7-H3 is produced by alternative splicing from the fourth intron of B7-H3 [18] or matrix metallopeptidase (MMP) [17], and the sB7-H3 serum level has been correlated with prognosis in various malignancies [19, 20].

## Receptor of B7-H3

**Fig. 2:**
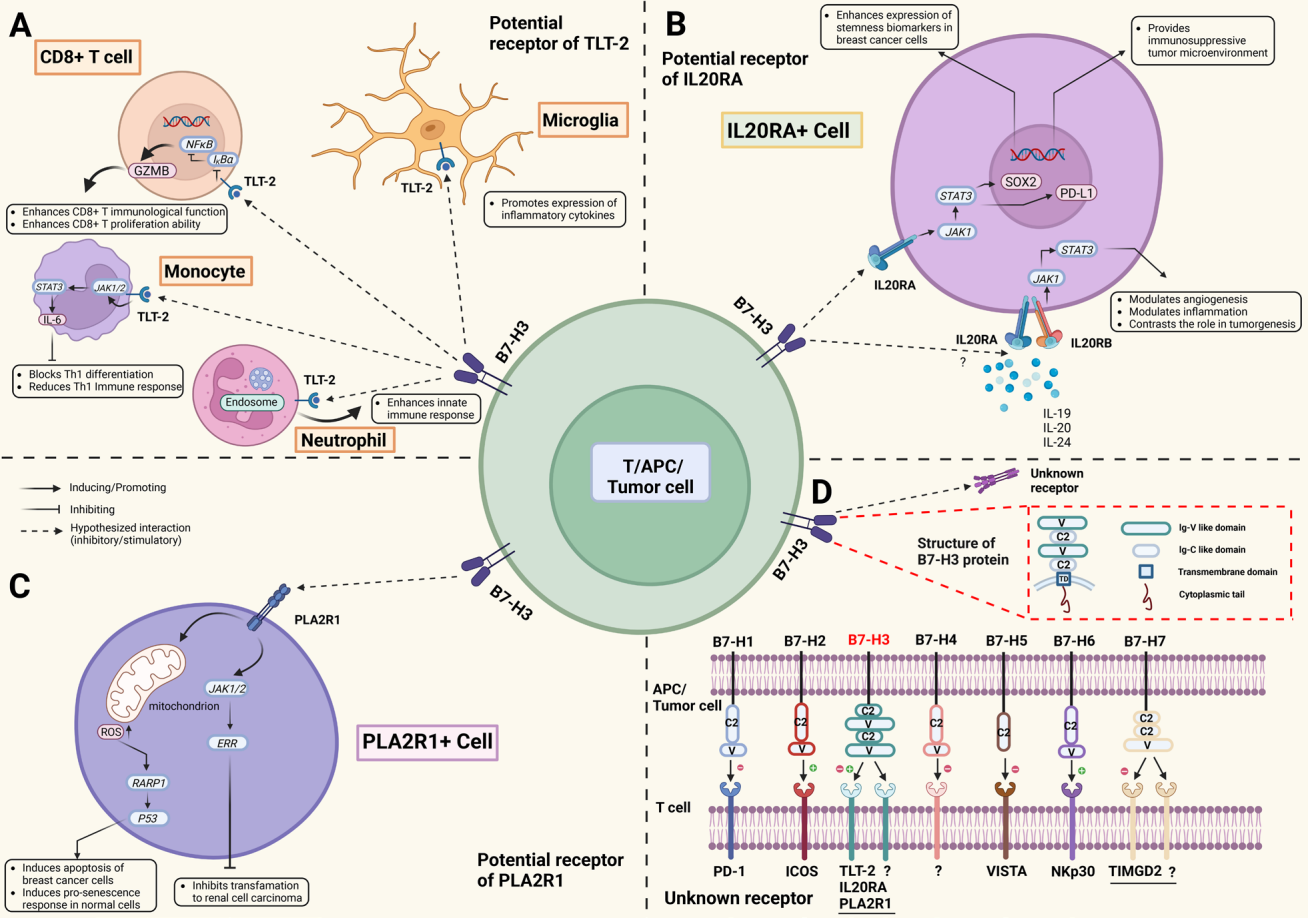
Structures, distributions, interactions and biological functions of B7-H3 and putative receptors. Three proteins have been identified as potential B7-H3 receptors, including TLT-2 (**A**), IL20RA (**B**) and PLA2R1 (**C**). TLT-2 is widely expressed on the surface of myeloid, B and T cells, and its function in specific cell types has been separately studied. TLT-2 plays a proinflammatory role in CD8+ T cells, neutrophils and microglia while reducing the Th1 immune response and blocking Th1 differentiation when activated on monocytes. The effect of B7-H3 binding to TLT-2 on CD8+ T cells is controversial, and the functional interaction between B7-H3 and TLT-2 in other cell types remains unknown (**A**). Little is known about the specific cell types that express IL20RA and PLA2R1 and their cell type-specific functions. IL20RA activation enhances breast cancer cell stemness and establishes an immunosuppressive TME via the JAK1/STAT3 signaling pathway, while modulation of the TME via the JAK1/STAT3 pathway through IL20RA and IL20RB is still disputed, requiring more robust and direct evidence (**B**). PLA2R1 has been indicated as a tumor-suppressive regulator that induces breast cancer cell apoptosis and inhibits transformation to renal cell carcinoma (**C**). Considering the diverse roles of B7-H3 in the TME, other unknown receptors must be reported continuously. Human B7-H3 gene locates in 15q24.1 and has 12 exons encoding 316 amino acids; the structure of B7-H3 (4IgB7-H3 here, the major isoform in humanity) comprises two identical pairs of extracellular lgV-like and IgC-like domains, a transmembrane region and a 45-aa cytoplasmic tail. Seven B7-H family members (B7-H1 to B7-H7) and their receptors expressed on T cells are also displayed, where B7-H3 binds to TLT-2, IL20RA, PLA2R1 and other interesting as yet unknown receptors. “+” in green indicates the immunostimulatory (positive) signal, and “−” in red indicates the immunosuppressive (negative) signal (**D**). TME, tumor microenvironment

The identity of the receptor of B7-H3 is controversial and has not been verified. The unknown nature of the B7-H3 receptor has become the biggest hurdle to understanding the biology of B7-H3, yet the available data for the B7-H3 receptor are still conflicting and scarce, although great efforts have been devoted to solving this issue. Figure 2 summarizes the structure, biological function and interaction with B7-H3 and putative B7-H3 receptors.

### TLT-2

Triggering receptor expressed on myeloid cells (TREM)-like transcript 2 (TLT-2, TREML2) was the first identified and most well-studied B7-H3 receptor candidate. TLT-2 is extensively expressed in neutrophils, macrophages, the B lymphoid lineage [21], microglia [22], CD8^+^ T cells and activated CD4^+^ T cells [23, 24]. Although the crystal structure of TLT-2 has not been solved, researchers have speculated that, similar to other members of the TREM family, TLT-2 is a single transmembrane protein in the immunoglobulin superfamily that contains a putative SH3 binding motif [25]. The function of TLT-2 has been widely studied in various components of the innate and adaptive immune systems. Ligation of TLT-2 by a monoclonal antibody activates neutrophils to induce reactive oxygen species production, degranulation and chemotaxis, especially in response to G protein-coupled receptor signaling [26] and TLT-2, whose expression in neutrophils is stimulated by inflammatory mediators, is predominantly localized in intracellular vesicles in neutrophils, potentially modulates the exocytosis process [27]. In microglia, TLT-2 promotes the expression of proinflammatory cytokines, antagonizing the anti-inflammatory role of TREM2 [28]. Using proteomics analysis and TLT-2 knockdown, Xu and colleagues revealed that TLT-2 activates NF-κB signaling by inhibiting IκBα to promote the expression of granzyme B (GZMB) and enhance the immune function and proliferation ability of CD8^+^ T cells [29]. However, in contrast to its overall proinflammatory effect on other cell types, TLT-2 expressed in monocytes promotes interleukin-6 (IL-6) expression via the Janus kinase (JAK)/signal transducer and activator of transcription 3 (STAT3) pathway, blocks Th1 differentiation and hinders the immune response to tuberculosis [30]. These studies suggest that TLT-2 is an important modulator of both the innate and adaptive immune systems. The multifaceted and seemingly contradictory function of TLT-2 as a putative B7-H3 receptor that is present in different cell types may potentially explain the contradictory role of B7-H3 in the immune response.

Using flow cytometry, Hashiguchi et al. found that the murine B7-H3 fusion protein B7-H3Ig specifically binds to TLT-2. The relative affinity between B7-H3Ig and TLT-2 was estimated to be comparable to the affinity between PD-L1 and PD-1 in the same model, and the B7-H3/TLT-2 interaction was shown to functionally enhance T cell responses and IL-2 and IFN-γ production [23], consistent with the first discovered role of B7-H3 [11]. The binding and flow cytometry analyses provided direct evidence for B7-H3 binding to TLT-2, although the difference between the B7-H3Ig fusion protein and endogenous membrane-bound B7-H3 and the difference between high TLT-2 expression in transfected cells and endogenous TLT-2 expression should not be ignored. In vivo validation was conducted in a murine contact hypersensitivity model [23] and tumor model [24], where both anti-B7-H3 and anti-TLT-2 monoclonal antibodies attenuated the inflammatory response. Unfortunately, the validation study still did not exclude the possibility that B7-H3 and TLT-2 stimulate the T cell response through different pathways. Later, Fang et al. found that sB7-H3 induces the chemotaxis of myeloid-derived suppressor cells (MDSCs) in vitro, which might be attenuated by blocking TLT-2 [31]. Together, these studies provide direct and indirect evidence for the hypothesis that TLT-2 is the receptor for B7-H3.

However, in another study by Leitner et al., no evidence was found to support the interaction between TLT-2 and B7-H3 in either a murine model or in human cells, in which B7-H3 also potently and consistently downregulated human T cell responses [32]. The authors performed an in-depth study that considered the ligand concentration, B7-H3 isoforms, types of fusion proteins and differences between murine and human genes. Although the analysis of the binding between murine B7-H3Ig and TLT-2 was very similar to the study by Hashiguchi et al. [23], differences in the cellular background, transfection efficiency and fusion protein construct might explain the discrepancy in the conclusions. Using a similar design to examine another cell line, CHO cells, Yan et al. did not detect any interaction between TLT-2 and B7-H3 [33]. Further studies provide other forms of evidence opposing the binding between these two molecules. The crystal structure of B7-H3 did not support the binding of TLT-2 and B7-H3 [16], and the unsynchronized expression of TLT-2 and B7-H3 might also serve as a negative indicator [29]. The binding of TLT-2 and B7-H3 is still disputed, further validation of the binding is needed, and studies exploring the functional interaction between B7-H3 and TLT-2 expressed in innate immune cells might also substantially improve our understanding of their interaction.

### IL20RA

With a new interactome platform using high-throughput data, Husain et al. found that interleukin-20 receptor subunit α (IL20RA) was the first target of B7-H3 binding [34]. The platform provided valuable information, as it detected the interaction between membrane proteins, consistent with the normal function of B7-H3. In the receptor library, all the single-pass transmembrane proteins in the public database were included to provide a comprehensive interactome landscape [34], while a previous study only screened proteins homologous to the CD28 family [23, 32]. The binding with IL20RA was further verified by Cao et al., who performed another extracellular vesicle-based high-throughput interactome platform [35], but the binding still requires further functional validation from either in vitro or in vivo studies.

IL20RA was first identified as a subunit of the binding complex that forms a heterodimer with interleukin-20 receptor subunit β (IL20RB) to bind IL20 [36]. IL20RA is a transmembrane glycoprotein containing two tandem β-sandwich domains in the extracellular domain and an intracellular domain that functions with its counterpart in the heterodimer, whose structure belongs to the type II interleukin receptor family [37]. The expression of IL20RA was enriched in many types of tissues, particularly in skin, lung and testis [36], but its expression was not detected in circulating immune cells, including monocytes, T cells, B cells and NK cells [38]. Further research on the IL20RA expression pattern, especially in the TME, is still lacking. As reviewed by Rutz et al., IL20RA transduces signals from IL19, IL20 and IL24 with IL20RB, activates JAK1/STAT3 pathways and modulates inflammation, angiogenesis, metabolism and epithelial remodeling, in which the signals from interleukin exert both tumor-promoting and tumor-suppressing effects [39]. The significance of IL20RA as a biomarker in cancer has been addressed [40], and overexpression of IL20RA alone promotes cancer stemness via the transcription factor SOX2 and induces an immunosuppressive TME by increasing PD-L1 expression [41]. Similarly, IL20RA activation might profoundly influence tumorigenesis, although the functional consequence of B7-H3 binding to IL20RA remains to be explored. As the available evidence suggests low expression of IL20RA in immune cells, the B7-H3-IL20RA interaction might indirectly modulate inflammation and the immune response through stromal and tumor cells, as shown in the study by Ungaro et al. that IL20RA promotes chronic inflammation via the lymphatic endothelium [42].

### PLA2R1

In addition to IL20RA, Cao and colleagues also detected phospholipase A2 receptor 1 (PLA2R1) as another high-affinity binding protein among all the single-pass transmembrane proteins with their extracellular vesicle-based interactome platform [35]. PLA2R1 belongs to the mannose receptor family, which is composed of a short cytoplasmic tail, a transmembrane domain, a tandem C-type lectin domain with 8 repeats, a fibronectin type II domain and a cysteine-rich terminal domain [43]. The expression pattern of PLA2R1 in normal cells has not been fully elucidated, but the decreased expression of PLA2R1 in different malignancies has been reviewed [44]. PLA2R1 has been shown to function as a tumor suppressor by inducing cellular senescence via the increased production of reactive oxygen species in mitochondria, suppression of PARP1 expression and activation of the p53 pathway in PLA2R1-overexpressing cells [45, 46], and it inhibited cell transformation into tumor cells via downstream estrogen-related receptor α1 and JAK2 signaling [47, 48]. On the other hand, the binding of secreted phospholipase A2, the known ligand for PLA2R1, increases the survival of mast cells [49] and promotes the migration of fibrosarcoma cells [50]. The mechanism by which the binding of B7-H3 to PLA2R1 modulates its antitumor function remains to be elucidated in the future.

The identity of the B7-H3 receptor is still uncertain, although three candidates, TLT-2, IL20RA and PLA2R1, have been proposed. The available evidence for these receptors does not fully explain the complicated effects of B7-H3 on malignant behaviors and the TME. As the B7 family member B7-1 binds to CD28, CTLA-4, PD-L1 and NGFR [51], B7-H6 interacts with KIR3DL3 to stimulate or TMIGD2 to inhibit the immune response [52]. The finding that B7-H3 has multiple binding partners must not be ignored, especially when considering the opposing roles of B7-H3. Recent interactome studies have expanded our scope of screening all the single-pass transmembrane proteins and identified two potential B7-H3 receptors that are not homologous to CD28. However, the evidence for the two recently discovered partners is far from sufficient, and further functional validation of IL20RA or PLA2R1 binding would be of great value. Unknown receptors with a structure beyond a single-pass transmembrane protein might also be discovered.

## B7-H3 and malignant behaviors

### B7-H3 in cancer proliferation

Unrestricted proliferation bypassing the cell cycle checkpoint is found in almost all tumor types and is a driving force of tumorigenesis [53]. Downregulation of B7-H3 reduced the proliferation of colorectal cancer (CRC) cell lines, and several key cell cycle-related proteins, including cyclin D1 and CDK4, were also dramatically decreased [54]. Recent studies have shown similar results in which B7-H3 overexpression significantly facilitated cell multiplication and migration in CRC cells [55]. B7-H3 silencing also reduced proliferation, invasion and migration in the A549 lung adenocarcinoma cell line [56]. In mouse spermatogonial stem cells (SSCs), B7-H3 was found to promote mouse SSC proliferation and cell cycle progression in a CCK-8 assay in which mouse SSCs were incubated with different concentrations of B7-H3 [57]. The proliferation promotion was inhibited by the PI3K inhibitor LY294002, indicating that B7-H3 promotes proliferation by activating the PI3K signaling pathway. Liu et al. found that B7-H3 binds to major vault protein (MVP) and activates MEK through the interaction between B-RAF and MEK in breast cancer stem cells, demonstrating a novel B7-H3/MVP/MEK signaling axis by which B7-H3 can promote cancer proliferation [58]. Both endogenous B7-H3 expression and exogenous B7-H3 stimulation promote cancer cell proliferation through various mechanisms.

### B7-H3 in deregulating cancer metabolism

Aberrant metabolism, including increased aerobic glycolysis and anabolic pathways, is a major hallmark of cancer that can fuel the tumorigenic process by providing energy, building blocks and redox potential [59]. Lim et al. confirmed in vivo and in vitro that B7-H3 overexpression promoted glucose intake and lactate production, contributing to aberrant glycolysis [60]. They further revealed that B7-H3 stabilized hypoxia inducible factor-1 (HIF-1α) through the transcription factor Nrf2 and its target genes SOD1, SOD2 and PRX3 and activated downstream glycolytic enzymes to exert a hyperglycolytic role. In ovarian cancer cell lines, B7-H3 knockout resulted in a decreased level of glycolysis and reduced the expression of lactate dehydrogenase A (LDHA), phosphoglycerate kinase 1 (PGK1) and HIF-1α, which suggests that B7-H3 promotes glycolysis [61]. Zuo et al. found that B7-H3 directly interacts with the rate-limiting glycolytic enzyme ENO1 and alters its activity in HeLa cells; moreover, B7-H3 silencing reduced the production of ATP, lactate, c-Myc and LDHA, indicating that B7-H3 alters metabolism by affecting the activity of ENO1 and the c-Myc-LDHA axis [62]. Li et al. explored the metabolism-reprogramming mechanism of B7-H3 in oral squamous carcinoma cells and demonstrated that B7-H3 upregulates the expression of HIF-1α, GlUT1 and PFKFB3 downstream through the PI3K/Akt/mTOR pathway to enhance glycolysis [63]. Shi et al. found that hexokinase 2 (HK2) was the key mediator of glucose metabolism regulation. They demonstrated that treating cells with HK2 inhibitors could reverse the B7-H3-induced increase in aerobic glycolysis, which suggested a novel underlying mechanism [64]. These studies strongly support the involvement of B7-H3 in the dysregulation of cancer cell metabolism and its contribution to tumorigenesis.

### B7-H3 in cancer invasion

B7-H3 has been reported to promote cancer cell migration and invasion in various types of cancer [55, 65, 66]. In glioma cells overexpressing B7-H3, Zhong et al. demonstrated that the JAK2/STAT3 signaling pathway was activated and that B7-H3-induced glioma progression was suppressed by a JAK2/STAT3 inhibitor. They further revealed that B7-H3 induces glioma invasion through the JAK2/STAT3/Slug/MMP-2/-9 pathway and is involved in epithelial‑mesenchymal transition (EMT) [67]. EMT is a key step in cancer metastasis. Yu et al. demonstrated that the downregulation of B7-H3 may inhibit EMT in lung adenocarcinoma cells [56]. In hepatocyte carcinoma, B7-H3 was found to promote EMT via the JAK2/STAT3/Slug pathway [68], while Liao et al. found another mechanism through which B7-H3 might promote EMT. They discovered that B7-H3 upregulated the expression of SIRT1 via the PI3K/AKT pathway in a non-small cell lung cancer (NSCLC) cell line and further promoted the expression of E-cadherin and EMT [69]. In clear cell renal cell carcinoma (CCRCC), B7-H3 was found to promote the EMT process in CCRCC cells by activating the PI3K/AKT and p38/ERK mitogen‐activated protein kinase (MAPK) signaling pathways, which are mediated by fibronectin [70]. Although mechanistically different, these studies demonstrate that B7-H3 greatly influences tumor invasion and metastasis.

### B7-H3 in cancer anti-apoptosis activity

B7-H3 can also promote tumor progression by inhibiting cancer cell apoptosis. In ovarian cancer cell lines, an evaluation of Annexin V-stained cells using flow cytometry showed that B7-H3 silencing promoted apoptosis mainly in the early stage, and subsequent western blotting results showed a decrease in the expression of the anti-apoptotic proteins Bcl-2 and Bcl-xl, as well as an increase in the levels of the proapoptotic proteins Bax, caspase-8 and cleaved caspase-8 [71]. Silencing of B7-H3 was also found to enhance apoptosis in cervical cancer cell lines [72]. The levels of phosphorylated JAK2 and STAT3 were increased in B7-H3-overexpressing cells, while treatment with the JAK2 inhibitor AG490 decreased the expression of related proteins in the JAK2/STAT3 pathway, and apoptosis was subsequently enhanced, indicating that B7-H3 exerts its anti-apoptotic effect through the JAK2/STAT3 pathway [71].

### B7-H3 in cancer therapy resistance

B7-H3 has been identified as promoting resistance to conventional cancer therapies in different types of cancer. It has been discovered that knockdown of B7-H3 increases the sensitivity of melanoma cells to the chemotherapeutic agents dacarbazine and cisplatin, which are small-molecule inhibitors targeting the MAPK and AKT/mTOR pathways [73, 74], and increases gemcitabine sensitivity in pancreatic carcinoma [75] and everolimus sensitivity in triple-negative breast cancer (TNBC) [76]. Altered glucose metabolism and increased apoptosis were shown to contribute to B7-H3-mediated chemotherapy resistance, which provides further evidence for the effect of B7-H3 on dysregulating metabolism and its anti-apoptotic role discussed previously [75, 76]. Notably, B7-H3 increased the radioresistance of gastric cancer cells by inhibiting baseline cell autophagy, apoptosis and DNA double-strand break repair [77]. Based on these studies, B7-H3 decreases the sensitivity of tumor cells to a series of chemotherapy agents and radiation and thus is a valuable target to augment the effect of conventional cancer therapy.

### B7-H3 in cancer stem cells

Cancer stem cells (CSCs) are a small subpopulation of cancer cells with “stemness” properties, and CSCs are widely accepted to promote metastasis, radioresistance, chemoresistance and cancer recurrence [78]. Significantly higher B7-H3 expression in CSCs than in the nonstem cell population have been observed in breast cancer, prostate cancer and head and neck squamous cell carcinoma (HNSCC) [58, 79, 80]. As demonstrated by Liu et al., after transfection with exogenous B7-H3, several breast cancer cell lines with B7-H3 overexpression dramatically enriched their CSC population, which was marked by CD24 and CD44, while B7-H3 knockdown led to the opposite results, and these effects were mediated via the B7-H3/MVP/MEK pathway [58]. B7-H3 has also been found to serve as an enrichment surface marker for Bmi1^+^ HNSCC CSCs, and anti-B7-H3 antibodies eliminated the CSC population and inhibited tumor growth in a CD8^+^ T-cell-dependent manner [80]. ALDH^+^ CD44^+^ prostate CSCs showed increased expression of B7-H3 after radiotherapy, and the expression difference between CSCs and bulk prostate cancer cells indicates that B7-H3 targeting immunotherapy is a promising combination alternative for prostate cancer therapy [79]. The association between stemness and B7-H3 expression provides support from another perspective for B7-H3-based antitumor therapy.

### B7-H3 and other cancer hallmarks

In a recent review, Hanahan et al. proposed several additional cancer hallmark traits, including phenotypic plasticity, senescent cells and epigenetic reprogramming [81]. There have been publications describing the involvement of B7-H3 in these malignant traits. B7-H3 unlocked phenotypic plasticity by blocking differentiation in alveolar rhabdomyosarcoma, where B7-H3 overexpression in alveolar rhabdomyosarcoma cell lines induced a myogenic differentiation block and a more invasive phenotype, while B7-H3 knockdown exerted the opposite effect [82]. B7-H3 also inhibits cellular senescence induced by doxorubicin (DOX) in CRC cell lines, possibly through the AKT/TM4SF1/SIRT1 pathway [83]. The epigenetic regulation of B7-H3 expression has been widely investigated. The expression level of B7-H3 was found by Wang et al. to be potentially regulated by the microRNA-29 family and B7-H3 promoter methylation [84]. In addition to microRNA-29, more than 10 microRNAs have been demonstrated to regulate the expression of B7-H3 and influence tumor behaviors, as reviewed by Feng et al. [9]. Moreover, N6-methyladenosine (m^6^A) RNA modification of B7-H3 mRNA was found to be significantly downregulated in CRC tissues compared with normal tissues, which further participated in immune escape, indicating epigenetic reprogramming’s role in B7-H3 function [85]. Altogether, B7-H3 is involved in tumor proliferation, metabolism, and invasion and a series of malignant behaviors, which confirms that B7-H3 is a valuable research object to further elucidate tumor biology and a therapeutic target to block tumor progression.

## B7-H3 in the tumor microenvironment

**Fig. 3:**
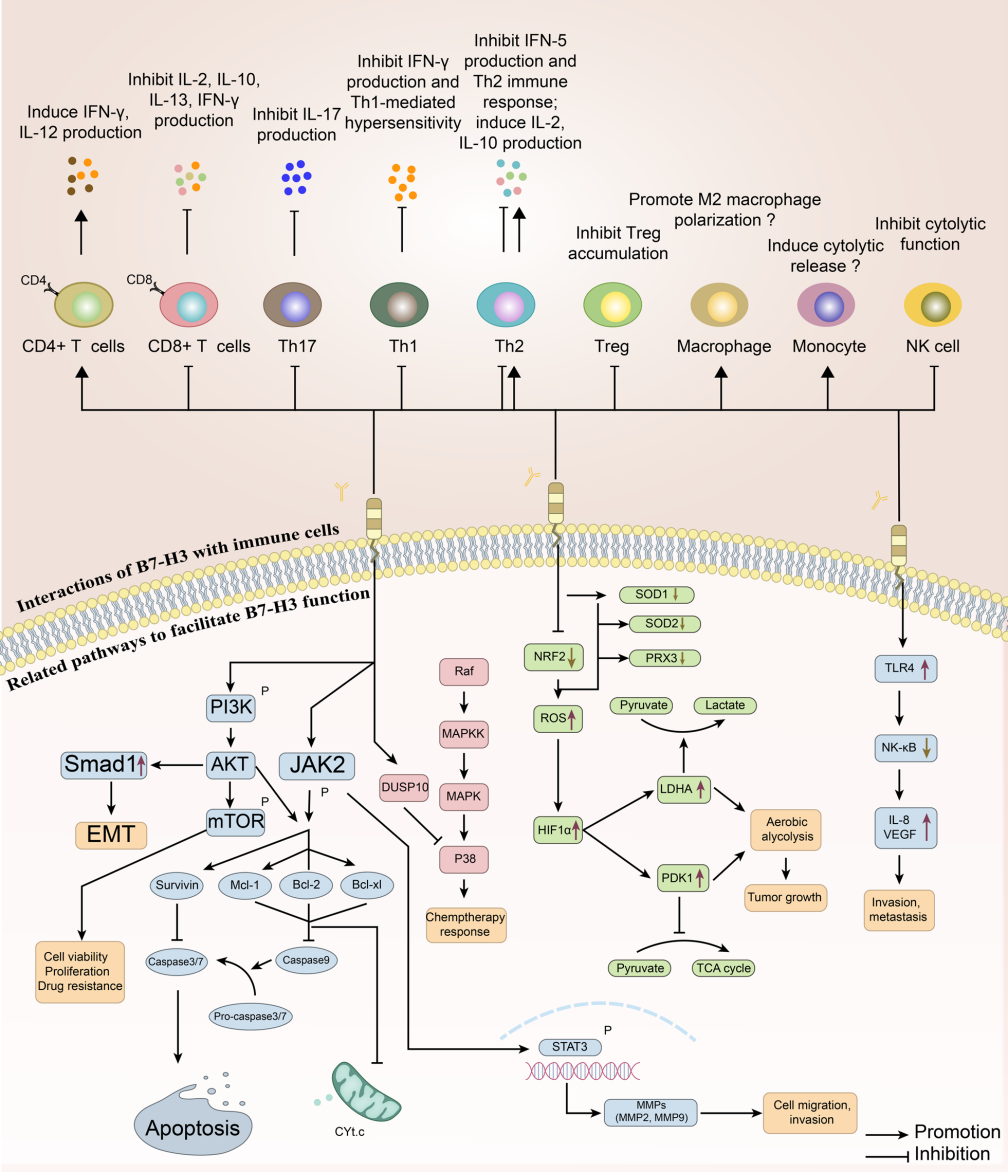
Interactions of B7-H3 with immune cells and related pathways facilitate B7-H3 function in the microenvironment. The top panel exhibits interactions with immune cells. B7-H3 was originally identified for its effect on promoting the growth of CD4+ T cells and inhibiting the growth of CD8+ T cells. Activated CD4+ T cells induce IFN-γ production and promote the production of IL-12, while IL-2, IL-10, IL-13 and IFN-γ production are suppressed in CD8+ T cells. B7-H3 also negatively regulates the release of IFN-γ and T cell proliferation in B7-H3-deficient mice. B7-H3 suppresses Th1- and Th2-mediated responses, activity and Treg accumulation. IFN-γ and IFN-5 production and Th1-mediated hypersensitivity are inhibited. However, the release of IL-2 and IL-10 is promoted from Th2 cells. B7-H3 enhances M2 macrophage polarization and the release of cytolytic factors from monocytes, which still requires stronger evidence. The cytolytic function of NK cells is curbed. The bottom panel presents distinct pathways to facilitate B7-H3 function. In the TME and related signaling pathways, the roles of B7-H3 are associated with tumor growth, migration, invasion, metastasis and other processes mediated by the PI3K/AKT/mTOR, JAK2/STAT3 and NF-κB signaling pathways and cell metabolism through the TCA cycle. Overall, B7-H3 regulates tumor cell invasion, migration, apoptosis, metabolism and drug response/resistance through classic pathways; B7-H3 also interacts with many types of immune cells in the microenvironment to influence the immune response. TME, tumor microenvironment; TCA cycle, tricarboxylic acid cycle

The tumor microenvironment (TME) dynamically modulates tumor progression and greatly influences the outcomes of cancer immunotherapy. The TME typically comprises immune cells, including tumor-infiltrating lymphocytes (TILs), tumor-associated macrophages (TAMs), dendritic cells (DCs) and natural killer (NK) cells; stromal cells; extracellular matrix (ECM) and secreted molecules, including cytokines, chemokines and exosomes; and blood and lymphatic vascular networks [86]. Accumulating evidence has been found to support the idea that B7-H3 modulates the immune response by influencing different TME characteristics. Figure 3 critically summarizes the interactions of B7-H3 with immune cells and related pathways to facilitate their functions.

### B7-H3 and TME immune cells

When discovered, B7-H3 was originally found to be an immune costimulator [11], where B7-H3-Ig induced the proliferation of CD4^+^ and CD8^+^ T cells, increased the secretion of interferon γ and enhanced cytotoxic T cell activity. The costimulatory role of B7-H3 was subsequently supported by several studies in different models, including cancer, autoimmune diseases and allografts [23, 87, 88]. However, in the past decade, most studies in the oncology field have indicated that B7-H3 plays an inhibitory role in the TME. The discrepancy in immunomodulatory roles is most evident between autoimmune diseases and cancer models. In contrast to the predominant immunoinhibitory data reported in tumor studies, B7-H3 plays a proinflammatory role in autoimmune disease models, with some controversies [89–91]. Several factors might contribute to the discrepancy in the immunomodulatory roles of B7-H3. The first is the disease-specific expression pattern of B7-H3. Aberrantly upregulated expression of B7-H3 has been detected in tumor cells, immune cells and a series of stromal cells in malignancies with different levels and distributions, while the expression of B7-H3 in synoviocytes, osteoblasts, endothelial cells and other cells was noted in an autoimmune disease model [92]. This difference in the expression pattern between disease models may reshape the immunomodulatory capacity of B7-H3. B7-H3 is expressed in not only adaptive immune cells but also cancer-associated fibroblasts, neutrophils, and the endothelium [93–95], where it may shape the immunosuppressive TME. The distribution and functions of these stromal cells vary substantially in different models, which might dominate the function of the most well-studied T cell-mediated immunoregulatory axis. The second is the multiple downstream pathways activated by different binding partners. As discussed in the receptor section, B7-H6 interacts with TMIGD2 to stimulate the immune response or with KIR3DL3 to inhibit the immune response [52], and B7 family members with multiple binding partners are not rare. The abundance of inhibitory B7-H3 receptors in certain TMEs compared with other models might at least partially explain the discrepancy. Despite the dispute in the overall conclusion, the immunomodulatory roles of B7-H3 mediated by interactions with different cellular components of the TME have been widely investigated.

The correlation between the function and differentiation of T helper cells and B7-H3 has mainly been explored in the setting of autoimmune diseases, with some controversy. Suh et al. found that B7-H3-deficient mice exhibited accelerated progression of experimental autoimmune encephalomyelitis [96] and produced higher concentrations of DNA autoantibodies [91]. In addition to the immunoinhibitory role of B7-H3, Suh et al. found that T helper cells in B7-H3-deficient mice preferentially differentiate into Th1 cells rather than Th2 cells, i.e., B7-H3 negatively regulates Th1 differentiation preferentially. On the other hand, in a model of allergic conjunctivitis, Fukushima et al. reported that B7-H3 negatively regulated both Th2 immune responses and Th1 immune responses [90]. Conflicting results were reported by Luo et al., who analyzed the roles of B7-H3 in regulating the Th1, Th2 and Th17 subsets in an autoimmune disease model, and the results suggested that B7-H3 has a costimulatory function for Th1/Th17 cells but a coinhibitory function in Th2 responses [89]. The seemingly contradictory effect of B7-H3 on these CD4^+^ T helper cells might result from the analysis of different disease settings and B7-H3 targeting approaches. Possible failure to block B7-H3 with antibodies due to the uncertainty of its receptor and possible cross-reaction with other B7 family members may hinder the discovery. The conclusion might also be different in the context of tumors; thus, further analysis of T helper cell differentiation in tumors might be helpful.

T lymphocytes are the major component of antitumor immunity, and a correlation between T cells inhibition and B7-H3 expression has been established in several cancer models. An early study revealed that B7-H3 inhibited T cell activity by downregulating the NF-κB, NFAT and AP-1 signaling pathways and that blocking B7-H3 enhanced T cell activation in murine models [97]. Recently, it was reported that blocking B7-H3 resulted in dramatically increased CD8^+^ T cell infiltration and subsequent tumor inhibition in HNSCC [80]. This inhibition behaves in a CD8^+^ T cell-dependent manner, with increased infiltration of NK cells and GZMB^+^ cells, which mediate the apoptosis of squamous cancer cells. In triple-negative breast cancer, NanoString results for tumor samples revealed that B7-H3 was overexpressed in samples from the low TIL group [98]. In ovarian cancer, B7-H3 was shown to be highly expressed in both tumor cells and TILs, and B7-H3 expressed in tumor cells has been shown to play the main role in immunity inhibition [99]. B7-H3 deficiency in a murine model significantly downregulated other coinhibitory molecules, including PD-1, and increased the production of the proliferation markers Ki-67, IFN-γ, TNF-α and granzyme B in CD8^+^ T cells, which indicated a role of B7-H3 in CD8^+^ T cell exhaustion. In the same model, CD4^+^ T cells and NK cells were found to shift into an active IFN-γ- and TNF-α-producing state in the TME [99]. It was also demonstrated in a murine NSCLC model that B7-H3 blockade led to an increased number and functional recovery of infiltrated CD8^+^ T cells [96]. The expression of B7-H3 was found to be critically correlated with nonresponsiveness to anti-PD-1 immunotherapy in patient-derived NSCLC samples, and dual blockade of PD-L1 and B7-H3 in a murine model revealed an enhanced antitumor effect, which highlights B7-H3 as a promising anti-PD-1 combination option.

Regulatory T (Treg) cells have been demonstrated to confer immune tolerance and are involved in cancer immune evasion [100]. It was established in an in vivo model that Treg cells affect DCs in situ, decrease MHC-II-peptide formation in DCs and induce the expression of IL-10 and B7-H3, subsequently rendering DCs immunosuppressive [101]. Reduced infiltration of Treg cells both in absolute number and in ratio was observed in a B7-H3-deficient model [99], and a significant positive correlation between the number of FOXP3+ Treg cells and B7-H3 expression has been identified in human NSCLC tissues [102], which indicates a possible immunosuppressive mechanism of B7-H3 mediated by the recruitment of Treg cells. Although B7-H3 expression was again found to be negatively related to CD8^+^ TILs and overall survival, no significant correlation was observed between B7-H3 expression and Foxp3+ Treg cells in a prostate cancer model [103]. Similarly, no significant correlation between B7-H3 expression and Treg cells was identified in breast cancer [104], indicating possible variations in TME characteristics in different TME settings, and more extensive investigation is needed.

TAMs are highly plastic cells that serve a multitude of functions and are one of the key components of the TME [105]. The activation states of TAMs are generally categorized into two types: M1 classically activated macrophages, which promote inflammation and serve as costimulatory molecules to enhance the T cell response, and M2 alternatively activated macrophages, which play a critical role in immune modulation and tumor progression [105]. In triple-negative breast cancer, B7-H3 was also found to be highly expressed in TAMs, and these B7-H3-high TAMs played great prometastatic and immunosuppressive roles through intriguing ECM reconstruction and tumor angiogenesis, eventually reducing T cell infiltration in the tumor microenvironment [106]. In murine ovarian cancer models, B7-H3 knockout tumor cells showed a reduced number of M2 macrophages and increased IFN-γ^+^ CD8^+^ T cell infiltration. CCL-2 production was found to be upregulated by B7-H3, potentially via the STAT3 pathway, and a downstream CCR inhibitor partly eliminated the effect of B7-H3 knockout on M2 macrophages, indicating that the B7-H3-CCL2-CCR2 axis modulates TAM function [107]. It was also found that B7-H3 upregulated by lncRNA NEAT1 promotes M2 macrophage polarization via the JAK2-STAT3 pathway in multiple myeloma [108], showing that TAMs are important mediators of the immune-inhibitory function of B7-H3.

Neutrophils are a prominent component of the innate immune system and are often found in the TME. In human gastric cancer, it was found that tumor-derived GM-CSF induces the proliferation of neutrophils and stimulates B7-H3 expression in neutrophils via the JAK2-STAT3 signaling pathway. B7-H3-high neutrophils, which are often found in the gastric cancer TME, are correlated with a poor prognosis and tumor progression in human gastric cancer [94]. DCs were also found to be correlated with B7-H3. In NSCLC tumor samples, DCs were found to highly express B7-H3, with reduced IL-12 secretion and T cell activation capacity [109], in accordance with a previous study in which bone marrow-derived DCs with high B7-H3 expression appeared to be highly immune-inhibitory [101].

The intense investigation into the mechanism by which B7-H3 influences immune cell function has connected B7-H3 expression to both adaptive and innate immune systems, in addition to the extensively studied immune checkpoint-mediated change in T cell function. The complex interactions between B7-H3 and multiple immune cells account for the complex immunoregulatory roles of B7-H3, whereas the immunoregulatory roles mainly depend on the specific TME cellular composition. Although B7-H3 was mainly identified as an inhibitory immunoregulator in cancers, its functions vary in different diseases and even in different types of cancer. The conclusions must be interpreted with caution when translating the result into other models. A cancer-type-specific analysis would be more informative.

### B7-H3 and vascular network

Aberrant angiogenesis is an important hallmark of cancer because it allows for the delivery of oxygen, nutrients, and growth factors and is even the route for tumor metastasis [110]. B7-H3 expression has been found in tumor-associated endothelial cells. Seaman et al. demonstrated that B7-H3 overexpression was often found in tumor endothelial cells, while normal angiogenic tissues were uniformly negative for this marker [111]. Using B7-H3 knockdown in human umbilical vein endothelial cells (HUVECs) and in vitro and in vivo Matrigel models, Lai et al. suggested that B7-H3 in HUVECs enhanced VEGF secretion and subsequently increased cell proliferation, migration and tube formation [95]. However, it was shown that in late endothelial progenitor cells (LEPCs), which are circulating vascular repair cells with abundant expression of B7-H3 on the cell surface, B7-H3 knockdown promotes endothelial cell differentiation and angiogenesis but inhibits proliferation and migration, indicating a complex role of B7-H3 in LEPCs [112]. In a CRC model, it was demonstrated that the NF-κB pathway has a major effect on B7-H3-induced VEGF-A expression in CRC cells [113]. In medulloblastoma (MB) cells, through F-actin visualization and angiogenesis tube formation assays, B7-H3-overexpressing MB cells were found to significantly promote the angiogenic ability of co-cultured HUVECs, which can be attenuated by miR-29, and in vivo chick chorioallantoic membrane angiogenesis assays demonstrated similar results [114]. Furthermore, they showed that B7-H3 overexpression upregulated a series of proangiogenic molecules, including IL-6, IL-1, VEGF-D and VEGFR2, and a significant correlation between MMP-9 levels and sB7-H3 levels was identified, suggesting that B7-H3 promotes angiogenesis by upregulating MMP-9 [114]. MMP-2 and B7-H3 have been shown to be correlatively upregulated in several tumor types, and in melanoma, B7-H3 silencing significantly reduced MMP-2 protein expression, as reviewed by Zhou et al. [115], indicating that MMP-2 is involved in B7-H3-mediated angiogenesis. In hepatocellular carcinoma (HCC), B7-H3 knockdown upregulated E-cadherin expression but inhibited AKT phosphorylation, VE-cadherin expression and MMP2/9 activation in HCC cell lines, suggesting a PI3K/AKT/MMP pathway for B7-H3-mediated MMP activation [116]. The expression of B7-H3 in both tumor cells and endothelial cells, as well as their crosstalk, fuels the process of ECM reconstruction and aberrant angiogenesis by inducing cytokine and MMP secretion.

### B7-H3 and other TME characteristics

**Fig. 4:**
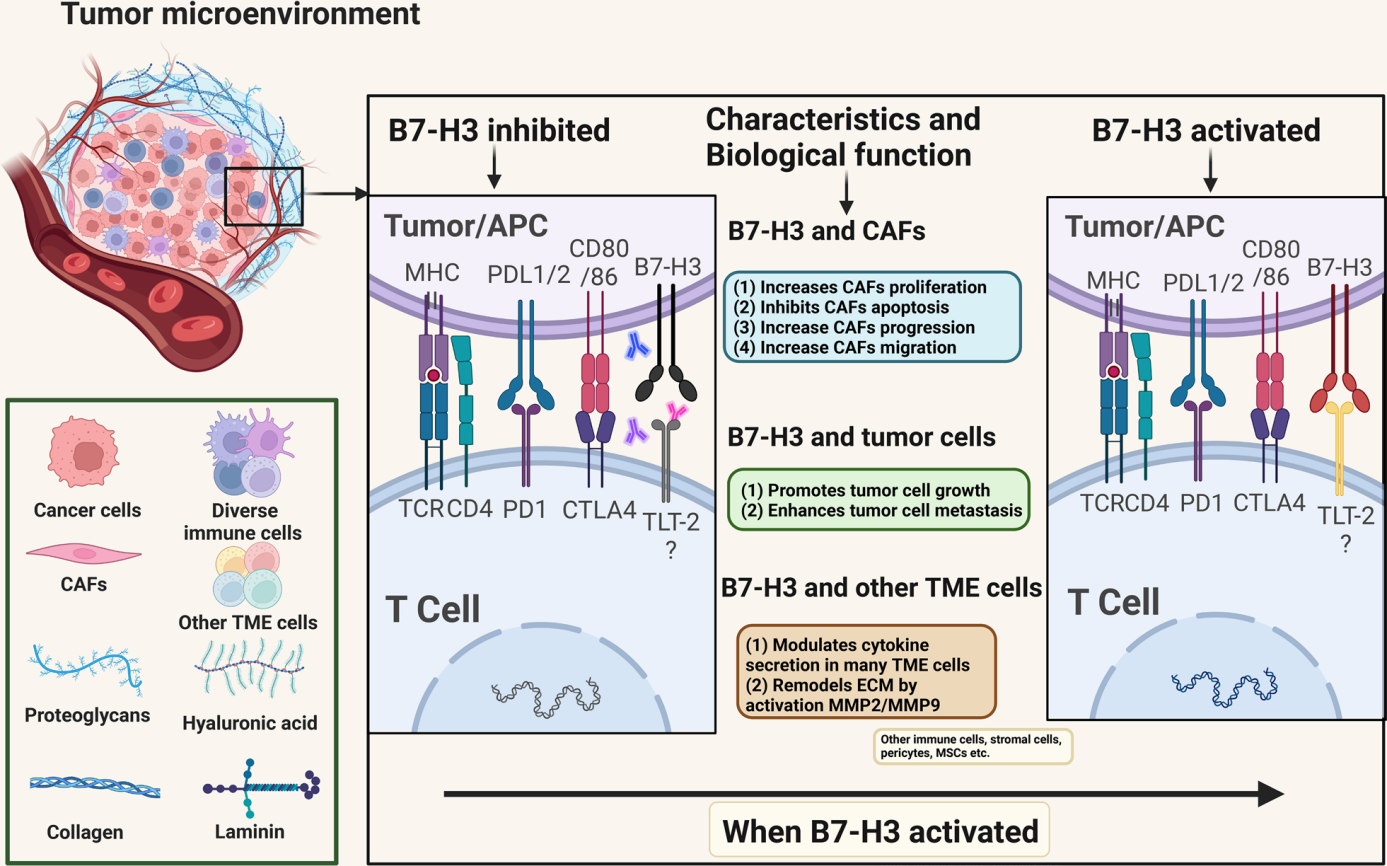
Main associations between B7-H3 and CAFs, tumor cells and other TME cells. Activated B7-H3 increases the proliferation, progression and migration of CAFs and inhibits the apoptosis of CAFs. Inhibiting B7-H3 in gastric cancer decreases the expression of IL-6, CXCL12, FGF1 and VEGF and suppresses the migration of CAFs. B7-H3 activation also promotes tumor cell growth and enhances tumor cell metastasis through AKT pathways. B7-H3 modulates cytokine secretion in many types of TME cells, including T cells, endothelial cells and CAFs, and B7-H3 helps to remodel the ECM by activating MMP2/MMP9. TME, tumor microenvironment; CAFs, cancer-associated fibroblasts; MSCs, mesenchymal stromal cells; ECM, extracellular matrix

Among all the stromal cells that populate the tumor microenvironment, cancer-associated fibroblasts (CAFs) are the most abundant and function in cell–cell contact, releasing numerous regulatory factors and remodeling the extracellular matrix [117]. Zhang et al. explored the relationship between B7-H3 and CAF function and revealed that B7-H3 knockdown in CAFs significantly inhibited cell proliferation, increased apoptosis, inhibited cell cycle progression and decreased the expression of hepatocyte growth factor protein and stromal cell-derived factor-1 protein, indicating that B7-H3 has a strong anti-apoptotic effect on CAFs [93]. They also found that B7-H3^+^ CAFs promoted renal cell carcinoma growth and metastasis both in vivo and in vitro, possibly through the AKT signaling pathway. In a gastric adenocarcinoma model, α-SMA and B7-H3 expression was detected in fibroblasts, and a positive correlation between their expression levels was found in stromal cells [118]. B7-H3 knockdown in gastric adenocarcinoma-derived CAFs caused decreased IL-6, CXCL12, FGF1 and VEGF expression and inhibited the migration ability of CAFs [118]. These results revealed that B7-H3 expression in CAFs increases their viability and secretory capacity to in turn promote the growth and metastasis of tumors, suggesting another B7-H3-mediated pro-tumorigenic interaction from B7-H3^+^ CAFs. It has also been widely demonstrated that B7-H3 modulates cytokine secretion in various TME cells, including T cells, endothelial cells and CAFs [11, 99, 114, 118], and B7-H3 is involved in ECM remodeling through the activation of MMP2/MMP9 [115, 119]. Overall, B7-H3 greatly shapes the TME in different ways. The main associations between B7-H3 and CAFs, tumor cells and other TME cells (i.e., immune cells, stromal cells, pericytes and mesenchymal stromal cells (MSCs)) are depicted in Fig. 4.

## B7-H3 in different malignancies

**Fig. 5:**
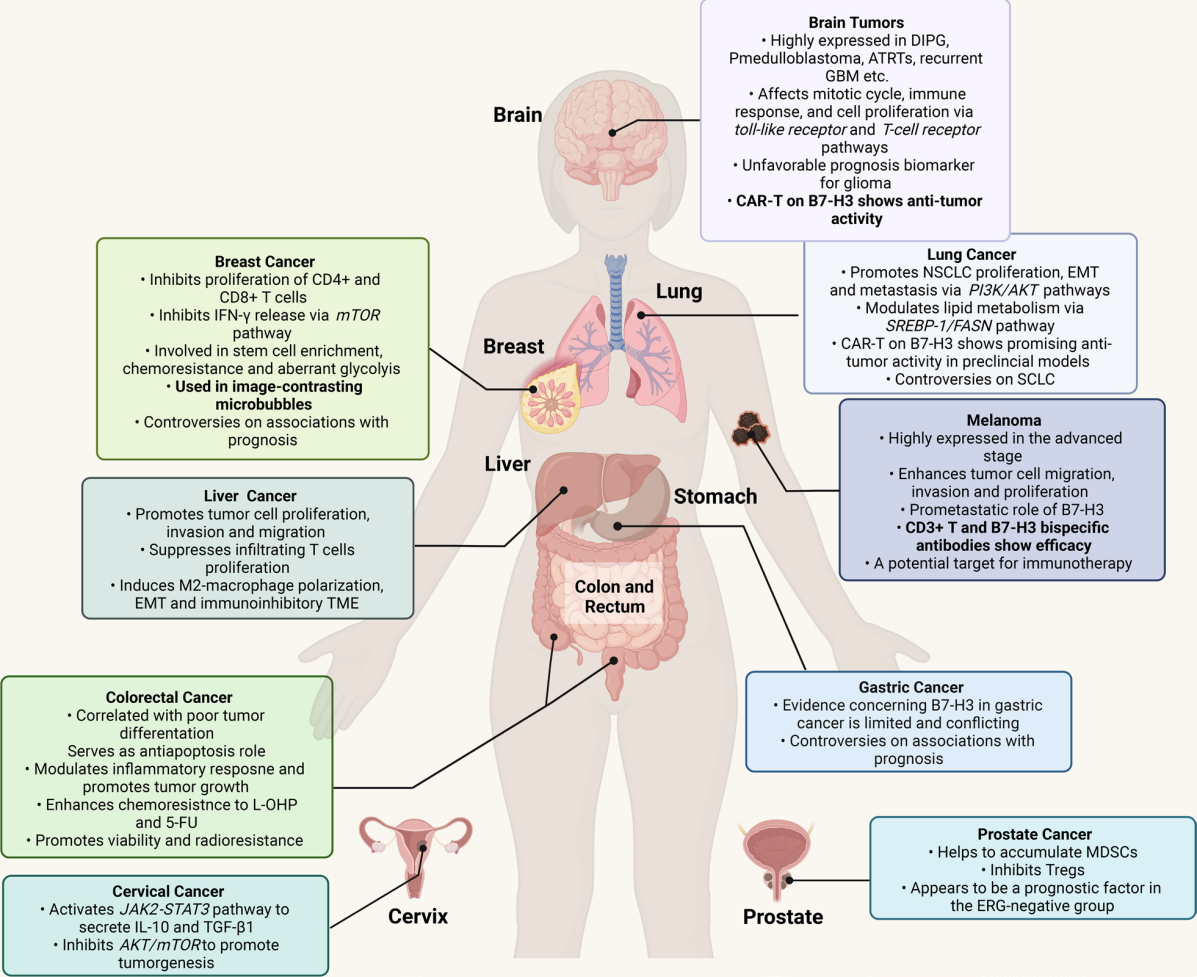
Roles of B7-H3 in several specific cancer types. B7-H3 has various roles in brain tumors, lung cancer, breast cancer, melanoma, liver cancer, gastric cancer, colorectal cancer, cervical cancer and prostate cancer by activating different mechanisms. B7-H3 is negatively associated with the prognosis of glioma and ERG-negative prostate cancer and serves as a promising immunotherapy target in brain tumors, lung cancer and melanoma. The boxes show the available characteristics and function of B7-H3 in cancers. Most characteristics are based on evidence from preclinical models, while specific characteristics highlighted in bold are based on evidence from human (clinical) studies/trials. DIPG, diffuse intrinsic pontine glioma; medulloblastoma, pediatric medulloblastoma; ATRTs, atypical teratoid/rhabdoid tumors; GBM, glioblastoma; CAR-T, chimeric antigen receptor-T cells; NSCLC, non-small cell lung cancer; EMT, epithelial–mesenchymal transformation; SCLC, small-cell lung cancer; MDSCs, myeloid-derived suppressor cells; Tregs, regulatory T cells; TME, tumor microenvironment

Immunohistochemical staining targeting B7-H3 in multiple normal tissues only revealed weak cytoplasmic staining in the salivary gland, gastric epithelium and adrenal gland [120], while B7-H3 has been found to be highly expressed in various cancer cells, as reviewed by Zhou et al. [121]. B7-H3 has been extensively studied in different cancer types, elucidating the expression level of B7-H3, its correlation with prognosis, and the possible underlying mechanism through which B7-H3 influences tumor progression. Here, we review the roles of B7-H3 in specific cancer types, and the main roles of B7-H3 are presented in Fig. 5.

### B7-H3 in lung cancer

B7-H3 has attracted strong interest in the field of lung cancer. B7-H3 expression was found in 510 out of 634 patients with NSCLC, and a high expression level was demonstrated to have a negative impact on prognosis [122]. On the other hand, in small cell lung cancer (SCLC), B7-H3 showed no significant correlation with clinicopathologic variables or TIL markers, although it was expressed in 64.9% of SCLC cases [123], while the contradictory conclusion that B7-H3 was a negative predictor in SCLC was suggested by Qiu et al. [124]. B7-H3 might promote NSCLC proliferation, EMT and metastasis via the PI3K/AKT pathway, as previously mentioned [56, 69]. It was also reported that downregulated B7-H3 can reduce lipid synthesis via the SREBP-1/FASN signaling pathway in lung cancer [125]. T cells expressing B7-H3-specific chimeric antigen receptors (CARs) and bispecific killer cell engager (BiKE)-redirected NK cells have shown significant antitumor activity in a preclinical model [126], and combined targeting of B7-H3 and PD-1 has resulted in promising response rates in clinical trials [127].

### B7-H3 in CRC

B7-H3 expression was detected in 50.8% of the primary CRC samples in a large cohort [128], and elevated B7-H3 expression was related to advanced overall stages, decreased disease-free survival and increased CD45RO T cell infiltration. In addition, Zhang et al. noted that higher B7-H3 expression was related to more lymph node involvement and poor tumor differentiation [129]. In CRC cells, it has been demonstrated that B7-H3 promotes tumor angiogenesis through the NF-κB pathway [113], and Meng et al. discovered that B7-H3 increases the expression of intracellular TNF-α, which modulates the inflammatory response and promotes tumor growth by inducing cell survival [130]. The anti-apoptotic role of B7-H3 exerted in a JAK2/STAT3-dependent manner was also verified in CRC cells [131]. In addition to different underlying mechanisms modulating tumorigenesis, correlations between B7-H3 and resistance to conventional cancer therapy have been extensively explored in CRC. B7-H3 has been shown to enhance chemoresistance to oxaliplatin (L-OHP) or 5-fluorouracil (5-FU) via the B7-H3-STAT3-HK2 axis [64] or in a STAT3-CDC25A-dependent manner [132]. B7-H3 expression was also found to be elevated after irradiation during radiotherapy for CRC, thus promoting cell viability and radioresistance via the B7-H3/KIF15/ERK axis [133]. Taken together, these studies thoroughly investigated the mechanism of B7-H3-mediated tumorigenesis, especially in CRC, and laid a solid foundation for ongoing trials evaluating B7-H3-targeted therapies for CRC.

### B7-H3 in breast cancer

Through transcriptome analysis, matched RNA and protein expression comparison and verification in 198 breast cancer samples, Kim et al. found that B7-H3 expression was significantly higher in tumor samples and highly expressed in 73.6% of the cases [134]. Although B7-H3 expression was negatively correlated with T cell infiltration and more frequently present in certain molecular subtypes, including TNBC, no significant relationship between overall survival (OS) and progression-free survival (PFS) was found in the study [134]. In contrast, Fang et al. reported that high B7-H3 expression was correlated with a poor prognosis, with a 56.8% B7-H3^+^ rate in 74 cases [135], and a B7-H3 association with the extent of regional nodal metastasis was also reported [136]. In a breast cancer cell line, B7-H3 expression was found to reduce the proliferation of CD4^+^ and CD8^+^ T cells and inhibit IFN-γ release via mTOR signaling [137], and modulating the TME through macrophages was another possible mechanism, as previously mentioned [106]. B7-H3 also regulates stem cell enrichment and promotes chemoresistance and aberrant glycolysis in breast cancer [58, 76]. In addition to a potential therapeutic target, B7-H3 also serves as a molecular ultrasound imaging target that can be used in image-contrasting microbubbles and has been applied in preclinical mammography [138]. The future applications of B7-H3 in breast cancer are promising and broad.

### B7-H3 in prostate cancer

Prostate cancer is the second most common cancer type in men [139]. Nunes et al. revealed a correlation between B7-H3 expression and worse outcome, especially the recurrence rate, in prostate cancer in two different cohorts, and a strong correlation between B7-H3 and androgen receptor (AR) protein expression was also revealed, although the B7-H3 expression rate was only 15% and 38% in the two cohorts [140]. In a large-scale immunohistochemistry analysis, B7-H3 immunostaining was positive in 47.0% of more than 17,000 prostate cancer cases, and B7-H3 appeared to be a negative prognostic factor, especially in the ERG-negative subgroup [141]. B7-H3 might promote prostate cancer progression through the accumulation of MDSCs [142], while a spontaneous prostate cancer model in mice revealed a contradictory costimulatory role of B7-H3 in inhibiting Treg cells [143]. B7-H3-targeting CAR T cell therapy has been assessed in murine prostate cancer stem cells and demonstrated a potent antitumor effect [79]. Both antibody-based and CAR-T therapies targeting B7-H3 in prostate cancer are being evaluated in clinical trials, which will be discussed further.

### B7-H3 in melanoma

Accumulating evidence has shown that melanoma responds well to immunotherapy, possibly due to the high immunogenicity of melanoma [144]. B7-H3 expression levels were revealed to be elevated in melanoma specimens, and higher expression was associated with advanced stages [145]. In vitro, B7-H3 enhanced cell migration and invasion via p-STAT3, although no significant effect on proliferation was observed in this study [145]. However, a later study demonstrated that B7-H3 overexpression enhanced proliferation and glycolytic capacity in melanoma cells, with reduced sensitivity toward dacarbazine, a MAPK- and AKT/mTOR-targeting small-molecule inhibitor [74]. Furthermore, Tekle et al. specifically elucidated a nonimmunological role of B7-H3: in melanoma cells, the expression levels of MMP-2, Stat3 and IL-8 were positively correlated with B7-H3 expression, while tissue inhibitor of metalloproteinase (TIMP)-1 and 2 showed the opposite results, indicating a prometastatic role of B7-H3 [146]. CD3^+^ T cell and B7-H3 bispecific antibodies and B7-H3-targeting CAR-T therapy have shown potent antimelanoma activity when investigated using in vivo and in vitro models [147, 148], validating the great potential of B7-H3 in melanoma immunotherapy. Nevertheless, mechanistic studies and preclinical success have not yet been translated into a clinical benefit, and a poor response rate was observed in patients with melanoma who were treated with anti-B7-H3 and anti-PD-1 antibodies [127]. Clinical evaluations of multiple modalities in a larger cohort are still ongoing.

### B7-H3 in gastric cancer

Multiple studies have shown that B7-H3 is widely present in gastric cancer and is associated with pathological features and prognosis. Wu et al. found that 58.8% of 102 gastric cancer tissues were B7-H3 positive and revealed a correlation between higher B7-H3 expression in cancer tissues and better overall survival, decreased tumor infiltration depth and more differentiated histological features, suggesting that B7-H3 is a positive indicator for gastric cancer prognosis [149]. In contrast, in stomach cancer cell lines and xenograft models, it was revealed that B7-H3 knockdown significantly inhibited cancer invasion and metastasis capacity [150]. Mechanistically, B7-H3^+^ neutrophils have been found in gastric cancer tissues, which increases tumor progression and is a negative predictive marker of reduced survival [94], and a more restricted CD8^+^ T cell location was noted in B7-H3-high gastric cancer samples, indicating a potential immunosuppressive role of B7-H3 in gastric cancer, although no significant survival difference was observed between the B7-H3-high and B7-H3-low groups in the study [151]. Evidence for the role of B7-H3 in gastric cancer is limited and conflicting, and more studies, particularly in patients with gastric cancer, are warranted.

### B7-H3 in liver cancer

The clinical significance of B7-H3 has been investigated in HCC. B7-H3 expression was found in 225 out of 240 HCC patients, and a correlation between high B7-H3 expression and poor survival and increased recurrence was confirmed in two independent cohorts [152]. Validation of the HCC cell line in vitro revealed that B7-H3 expression promoted cell proliferation, invasion and migration and suppressed the proliferation and IFN-γ secretion of infiltrating T cells [152, 153]. It has been demonstrated that B7-H3 promotes EMT and HCC invasion via the JAK2/STAT3/slug pathway [68], and it was discovered that B7-H3 induces M2 polarization of TAMs and contributes to an immunoinhibitory TME in a STAT3-dependent manner [154]. The diagnostic potential of serum sB7-H3 in early-stage hepatocellular carcinoma has also been investigated, and a promising result was found [155].

### B7-H3 in cervical cancer

B7-H3 expression was found in 62.8% of 673 cervical carcinomas or adenocarcinomas, and shorter disease-specific survival was found in the B7-H3-expressing group [156]. The increase in the secretion of IL-10 and TGF-β1 via the JAK2-STAT3 pathway activated by B7-H3 has been suggested as an underlying tumor-promoting mechanism [157], and AKT/mTOR might also be involved in tumorigenesis, as it was inhibited in SiHa cervical cancer cells by B7-H3 overexpression [158]. Although the significance of B7-H3 expression and its pro-tumorigenic mechanism has been investigated, implementation of B7-H3 immunotherapy even in preclinical cervical cancer models is not currently available.

### B7-H3 in brain tumors

B7-H3 has been reported to be extensively expressed in diffuse intrinsic pontine glioma [159], pediatric medulloblastoma [160], atypical teratoid/rhabdoid tumors (ATRTs) [161], recurrent glioblastoma [162] and gliomas [84]. Based on gene ontology analysis in a public database, B7-H3 was found to be involved in Toll-like receptor signaling and T cell receptor signaling, affect the mitotic cycle, immune response and cell proliferation, and serve as an unfavorable prognostic marker in glioma [84]. In GBM, the two different isoforms of B7-H3 appeared to function differently, in which 4IgB7-H3 expression was restricted in GBM cells and can serve as a target for GBM-targeting therapy, whereas 2IgB7-H3 expression was higher in GBM recurrences and increased resistance to temozolomide [162]. Chimeric antigen receptor (CAR) T cells targeting B7-H3 have shown potent antitumor activity in xenograft murine models of ATRTs, glioma and glioblastoma [161, 163, 164]. B7-H3 provides a promising target therapy in brain tumors and is being intensively evaluated in clinical trials. CAR-T therapy and radionucleotide-based antibody therapy showed preclinical benefits. Issues related to delivery, toxicity and sustainability of B7-H3-targeted therapeutics in the central nervous system remain to be addressed in clinical trials [165]. Table 1 further summarizes the role of B7-H3 in other cancer types not mentioned above [99, 166–172].

**Table 1:** B7-H3 in other cancers

Cancer types	Authors	Year	Type of samples analyzed	Expression	Functions	References
Esophageal cancer	Chen et al.	2015	Cancer cell line and human esophageal cancer tissues	High B7-H3 expression in 97 out of 174 cancer tissues, correlated with poorer survival and invasion depth	B7-H3 expression inversely correlated with infiltrated T cells	[166]
Merkel cell carcinoma	Aung et al.	2019	MCC cancer tissues	Consistent low expression of B7-H3 in MCC tumor cell, 9/20 strongly expressed in associated endothelial cells	Colocalization of endothelia CD31 expression and B7-H3 expression is a poor prognostic indicator	[167]
Ovarian cancer	Cai et al.	2020	Ovarian cancer xenograft model	B7-H3 robustly expressed in 24 ovarian cancer tissues	B7-H3 inhibits CD8^+^ T cell function, correlates with T cell exhaustion, which can be reversed by targeting B7-H3 in murine model	[99]
Acute myeloid leukemia	Lichtman et al.	2021	AML cell lines, primary AML blast, normal bone marrow and xenograft models	B7-H3 is highly expressed on monocytic AML cell lines and primary AML blasts from patients	B7-H3 CAR-T showed potent anti-AML effect in vitro and in xenograft model	[168]
Pancreatic cancer	Si et al.	2022	Pancreatic adenocarcinoma tissues	High B7-H3 expression in 52/104 cases, verified in TCGA data	High B7-H3 expression correlates with poor differentiation level, predicts a poor prognosis	[169]
Urothelial carcinoma	Koyama et al.	2020	Urothelial carcinoma tissues	B7-H3 expression was observed in 36 of 271 (13%) cases	B7-H3 expression was significantly associated with shorter PFS, disease-specific survival, tumor stage, grade and lymph node involvement	[170]
Laryngeal squamous cell cancer	Li et al.	2021	LSCC tissues and adjacent tissues	75/122 (61.5%) stained strongly positive for B7-H3, verified by RNA seq and TCGA data	B7-H3 overexpression significantly correlated with poor OS and lower TIL density	[171]
Skull base chordoma	Long et al.	2021	Chordoma samples and B7-H3 positive cell lines	B7-H3 positively was observed in 7 out of 45 (16%) chordoma samples	B7-H3 CAR-T showed potent anti-chordoma effect in vitro	[172]

(Not all clinical studies were included in this table due to the space limitation)

Among different tumor types, B7-H3 is generally discovered to induce an inhibitory TME and malignant traits, and it represents an unfavorable prognostic marker, while controversies remain in a variety of tumor types, including SCLC, gastric cancer, and prostate cancer. Theoretically, cancer types with firm and substantial support from preclinical models, a high B7-H3 expression rate, and a “hot” immune landscape, such as NSCLC and melanomas, seem to have greater potential to respond to B7-H3 immunotherapy [173]. Currently available results from early-phase clinical trials have shown the potential of B7-H3 immunotherapy mainly in treating NSCLC and brain tumors, with efficacy in multiple cancer types still being evaluated. Additional results obtained from clinical trials will validate the preclinical conclusions and broaden our understanding of B7-H3-targeted therapy.

## Immunotherapy targeting B7-H3

**Fig. 6:**
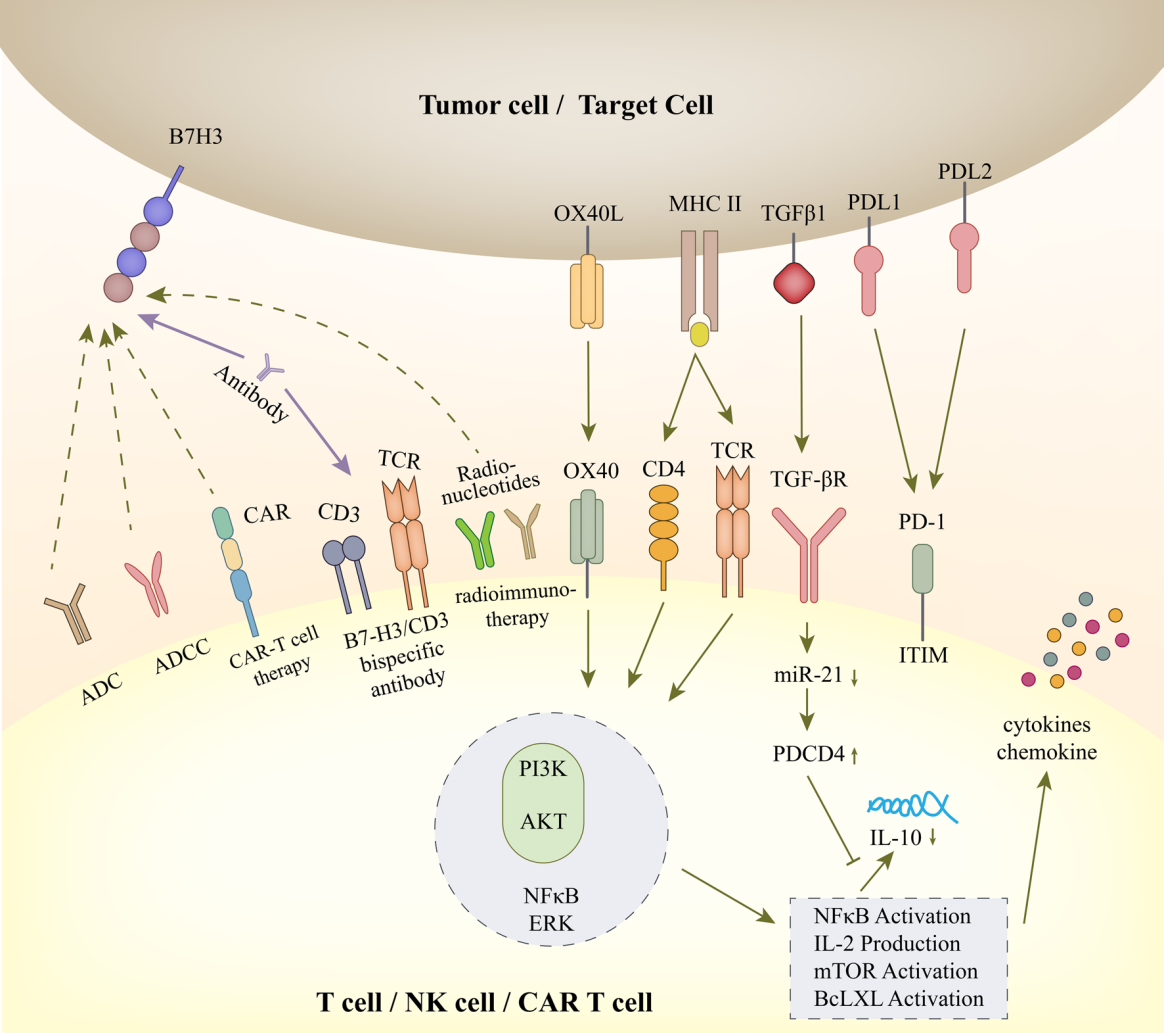
Immunology and future clinical immunotherapy of B7-H3. In T cells, OX40 liganded by OX40 L and the CD4/TCR-MHC II-antigen peptide complex elicit the following signals with a number of well-established proinflammatory mediators, such as PI3K, AKT, NFκB and ERK. Through the PI3K/AKT pathway, many downstream signatures are activated, including NFκB, IL-2 production, mTOR activation and Bcl-xl activation. Then, activated NFκB stimulates the release of cytokines and chemokines. The activation of the TGFβ receptor can inhibit the maturation of miR-21 and enhance PDCD4 levels. The translation of the anti-inflammatory cytokine IL-10 is suppressed in this signal, and the level is downregulated. TGFβ1 can participate in the adhesion, migration and invasion of renal cell carcinoma (RCC) cells. Clinical immunotherapy targeting B7-H3 includes blockade of B7-H3 monoclonal antibodies (mAbs), although the ligand is unclear; B7-H3-specific antibody‒drug conjugates (ADCs); B7-H3-specific antibody-dependent cell-mediated cytotoxicity (ADCC); B7-H3 and CD3 bispecific antibodies; engineered chimeric antigen receptor T cells (CAR-T cells); radionucleotides-induced radioimmunotherapy; other combined therapies (combined with PD-L1, PD-L2, etc.)

Although the receptor for B7-H3 has not been identified and the pro-tumorigenic role of B7-H3 has not been fully elucidated, success has been achieved in suppressing tumor growth by targeting B7-H3 as an inhibitory immune checkpoint in preclinical models and has greatly kindled the enthusiasm for clinical translation. Treatments targeting B7-H3 with different modalities are being intensely evaluated in clinical trials. Current immunotherapies strategies targeting B7-H3 are diagrammed in Fig. 6. Ongoing clinical trials targeting B7-H3 are summarized in Table 2, while the currently available clinical outcomes of these approaches are presented in detail in Table 3.

**Table 2:** Ongoing clinical trials targeting B7-H3

Trial number	Intervention	Intervention type	Phase	Status	Cancer type
NCT04145622	DS-7300a	B7-H3 ADC	I/II	Recruiting	Advanced solid tumors
NCT05280470	DS-7300a	B7-H3 ADC	II	Recruiting	Extensive-stage small cell lung cancer
NCT03729596	MGC018/ MGA012	Anti-B7-H3 ADC with Anti-PD-1 Ab	I/II	Recruiting	6 advanced solid tumors
NCT02192567	DS-5573a	B7-H3 ADCC	I	Terminated	Advanced solid tumors
NCT01391143	MGA271	B7-H3 ADCC	I	Completed	Prostate cancer, melanoma, renal cell carcinoma, triple-negative breast cancer, head and neck cancer, bladder cancer and NSCLC
NCT02982941	MGA271	B7-H3 ADCC	I	Completed	Solid tumor
NCT02923180	MGA271	B7-H3 ADCC	II	Active, not recruiting	Prostate cancer
NCT02381314	MGA271/Ipilimumab	Anti-B7-H3 ADCC with Anti-CTLA-4 Ab	I	Completed	Melanoma and NSCLC
NCT04630769	MGA271/ FT516 and IL2	Anti-B7-H3 ADCC with NK cell enhancing	I	Recruiting	Ovarian cancer
NCT04634825	MGA271/MGA012/MGD013	Anti-B7-H3 ADCC with Anti-PD-1 Ab or PD-1 X LAG-3 BiAb	II	Terminated	Head and neck cancer
NCT02475213	MGA271/pembrolizumab/MGA 012	Anti-B7-H3 ADCC with Anti-PD-1 Ab	I	Completed	Melanoma, head and neck cancer, NSCLC, urothelial carcinoma
NCT02628535	MGD009	B7-H3 X CD3 BiAb	I	Terminated	Mesothelioma and 11 other cancers
NCT03406949	MGD009/ MGA012	B7-H3 CD3 BiAb with Anti-PD-1 Ab	I	Active not recruiting	Advanced solid tumors
NCT04432649	4SCAR-276	CAR T cells	I/II	Recruiting	Solid tumor
NCT05143151	CD276 CAR-T	CAR T cells	I/II	Recruiting	Advanced pancreatic carcinoma
NCT04637503	Combined 4SCAR-276	CAR T cells	I/II	Recruiting	Neuroblastoma
NCT04864821	B7-H3 CAR-T	CAR T cells	I	Not yet recruiting	4 solid tumors
NCT04185038	SCRI-CARB7H3	CAR T cells	I	Recruiting	Central nervous system tumors
NCT04691713	B7-H3 CAR-T	CAR T cells	Not applicable	Recruiting	Solid tumor
NCT04385173	B7-H3 CAR-T	CAR T cells	I	Recruiting	Recurrent/refractory glioblastoma
NCT04670068	B7-H3 CAR-T	CAR T cells	I	Recruiting	Recurrent epithelial ovarian cancer
NCT04483778	4-1BBζ B7H3-EGFRt-DHFR	CAR T cells	I	Recruiting	Recurrent/refractory solid tumors
NCT04077866	B7-H3 CAR-T	CAR T cells	I/II	Recruiting	Recurrent/refractory glioblastoma
NCT05241392	B7-H3 CAR-T	CAR T cells	I	Recruiting	Glioblastoma
NCT04897321	B7-H3 CAR-T	CAR T cells	I	Recruiting	Pediatric solid tumor
NCT05211557	fhB7-H3 CAR-T	CAR T cells	I/II	Recruiting	Recurrent ovarian cancer
NCT03198052	B7-H3 and 10 other CAR-T	CAR T cells	I	Recruiting	Lung cancer
NCT04842812	B7-H3 and 11 other engineered CAR-T	CAR T cells	I	Recruiting	Advanced solid tumors
NCT05190185	TAA06	CAR T cells	I	Recruiting	Malignant melanoma, lung cancer, or colorectal cancer
NCT04692948	TAA06	CAR T cells	Not applicable	Recruiting	Acute myeloid leukemia
NCT01502917	124I-omburtamab	Radioimmunotherapy	I	Terminate	Brain cancer
NCT00089245	131I-omburtamab	Radioimmunotherapy	I	Active, not recruiting	Neuroblastoma, sarcoma and CNS tumors
NCT01099644	131I-omburtamab	Radioimmunotherapy	I	Active, not recruiting	Peritoneal cancer
NCT03275402	131I-omburtamab	Radioimmunotherapy	II/III	Recruiting	Neuroblastoma, CNS and leptomeningeal metastases
NCT05063357	131I-omburtamab	Radioimmunotherapy	I	Not yet recruiting	Diffuse intrinsic pontine glioma
NCT04022213	131I-omburtamab	Radioimmunotherapy	II	Recruiting	Peritoneum solid tumors
NCT04743661	131I-omburtamab	Radioimmunotherapy	II	Active, not recruiting	Recurrent medulloblastoma and ependymoma
NCT04315246	177Lu-DTPA-omburtamab	Radioimmunotherapy	I/II	Recruiting	Leptomeningeal metastasis solid tumor
NCT04167618	177Lu-DTPA-omburtamab	Radioimmunotherapy	I/II	Recruiting	Medulloblastoma

**Table 3:** Currently available clinical outcomes of B7-H3 targeting immunotherapies

Agent	Agent type	Trial ID	Type of study	Cancer types	Enrollment	Study design	Main conclusion	References
MGC018	ADC	NCT03729596	Phase I/II clinical trial	HNSCC, TNBC, melanoma, NSCLC, metastatic castrate-resistant prostate cancer (mCRPC)	80	Open-label dose escalation + cohort expansion	49/80 of the enrolled pts took 3 mg/kg MGC018 as determined in dose escalation study. 87.7% encountered at least 1 adverse event, most commonly neutropenia, fatigue, palmar-plantar erythron dysesthesia and headache. PSA decline and tumor regression was observed in prostate cancer pts	[176, 177]
DS-7300	ADC	NCT04145622	Phase I/II clinical trial	11 advanced solid cancers	127	Open-label dose escalation + cohort expansion	Treatment-emergent adverse events occurred in 124/127 pts (98%); the most common were nausea (61%), infusion-related reaction (35%), and vomiting (31%). Responses were observed in 30/91 evaluable pts (33%) in total, 7/9 pts with SCLC, 2/5 with NSCLC, and 16/42 with mCRPC	[179]
MGA271	ADCC	NCT01391143	Phase I clinical trial	7 advanced solid cancers	46	Open-label single-arm	MGA271 was well tolerated, with no dose-limiting toxicity. Pts experienced disease stabilization (> 12 weeks) and tumor shrinkage (2–69%) across several tumor types	[182]
MGA271	ADCC	NCT02923180	Phase II clinical trial	Prostate cancer	32	Open-label single-arm	12% of the enrolled pts experienced grade 3/4 adverse events. Post-treatment PSA declines, PSA0 at 1-year post-op, Gleason grade group changes showed promising results. Pathologic and immunologic evaluation of prostate revealed upregulation of CD8+ T cells, PD-1/PD-L1 expression, and immune activation	[183]
MGA271 + pembrolizumab	ADCC	NCT02475213	Phase I/II clinical trial	Advanced solid tumors	133	Open-label dose escalation	116/133 pts experienced treatment-related adverse events, 38/133 ≥ grade 3. Objective response was observed in 6/18 pts with HNSCC and in 5/14 pts with NSCLC, 1/17 patients with urothelial cancer and 1/13 pts with melanoma. Pts with previous ICI treatment had a poorer prognosis	[127]
B7H3 CAR-T	CAR T cells	ChiCTR1900023435 ChiCTR2100044386	Case reports	Meningioma, glioblastoma and basal cell carcinoma	–	Case reports	CAR-T infusion was well tolerated in three cases. No obvious therapy response was observed in meningioma and glioblastoma cases. Partial response in basal cell carcinoma case	[196–198]
4-1BBζ B7H3-EGFRt-DHFR	CAR T cells	NCT04483778	Phase I clinical trial	Relapsed/ refractory non-CNS tumors in pediatric or young adult patients	16	Open-label non-randomized two arms	No dose-limiting toxicity was observed in the first infusion, maximum circulating CAR-T expansion on first infusion was 4.98 cells/μL with median persistence of 28 days. Stable disease was observed in 3 of the 9 pts infused	[199]
131I-Omburtamab	Radioimmunotherapy	NCT00089245	Phase I clinical trial	Recurrent or metastatic neuroblastoma	105	Open-label single-arm	Self-limited fever, nausea and headache, creatinine elevation, and grade 1 and 3 transient elevated serum transaminase was observed. Nearly 50% of pts survive at least 36 months. Over 50% of pts were still alive when data was last updated	[202, 203]
131I-Omburtamab	Radioimmunotherapy	–	Clinical retrospective analysis	Recurrent rhabdomyosarcoma	23	Retrospective review	A prolonged survival of pts receiving intraventricular 131I-Omburtamab (*P* = 0.003)	[205]
124I-Omburtamab	Radioimmunotherapy	NCT01502917	Phase I clinical trial	Diffuse intrinsic pontine glioma in children	46	Open-label dose escalation	10/46 enrolled pts experienced grade 3 adverse events considered related to the agent. A dose up to 8 mCi and infusion volume of 8 mL were found to be safe. The median survival increased 3–4 months compared to historical control data	[207, 208]

### Targeting B7-H3 with an ADC

Antibody–drug conjugates (ADCs), which consist of a humanized antibody to target tumors, a potent cytotoxic payload and a linker to connect them, are a novel approach for cancer therapy [174]. MGC018, a developing ADC with a duocarmycin payload, has shown promising antitumor activity in preclinical models of breast, ovarian, prostate, lung cancer, head and neck cancer as well as melanoma, with bystander killing effect to eradicate tumors heterogeneously expressing B7-H3 [175]. MGC018 in six advanced solid tumors is being evaluated in a phase I/II clinical trial (NCT03729596); dose escalation study found a generally acceptable safety profile with two dose-limiting toxicities: one grade 4 neutropenia and one grade 3 fatigue [176]. Eighty patients were enrolled for cohort expansion, 87.7% of the patients encountered at least 1 adverse event, among which neutropenia, fatigue, palmar-plantar erythron dysesthesia and headache were seen > 10% of the patients. While further evaluation is still on the way, prostate-specific antigen (PSA) decline and tumor regression has been observed in prostate cancer patients [177].

DS-7300a is another B7-H3 targeting ADC which contains a DNA topoisomerase I inhibitor payload DXd and exerts potent antitumor activities in preclinical models [178]. The safety and efficacy of DS-7300a are being investigated in NCT04145622, and recently released interim results showed DS-7300a was well tolerated in heavily treated advanced tumor patients, and the objective response was observed in 30 out of 91 evaluable patients [179]. The early success of DS-7300a has greatly motivated the researchers and another trial specifically analyzing DS-7300a's efficacy in SCLC has been launched recently (NCT05280470).

### Targeting B7-H3 via ADCC

Antibody-dependent cellular cytotoxicity (ADCC) relies on the interaction between the Fc portion of an antibody and immune cells to eradicate targets [180]. MGA271 (enoblituzumab), which is a humanized IgG1 B7-H3 targeting antibody developed by Loo et al., contains a five amino acid change at its humanized Fc site for increased activation affinity and showed potent antitumor activity in renal cell carcinoma and bladder cancer xenograft models [181]. Thus, MGA271 is being extensively evaluated in clinical trials (NCT02982941, NCT02923180, NCT02381314, NCT04630769, NCT04634825 and NCT02475213). Interim data from NCT01391143 showed an acceptable safety profile of MGA271 in patients with B7-H3^+^ tumors, where patients experienced disease stabilization or tumor shrinkage across several tumor types [182]. In a phase II single-arm trial evaluating the neoadjuvant use of MGA271 (NCT02923180), 32 patients with prostate cancer were enrolled and received neoadjuvant MGA271 50 days prior to prostatectomy. Twelve percent of the enrolled patients experienced grade 3/4 adverse events, post-treatment PSA declines (> 10%) were observed in 31% of the patients and PSA0 at 1-year post-op was seen in 66% of the patients. Gleason grade group changes were significantly associated with MGA271 treatment compared to matched historical controls. Pathologic and immunologic evaluation of the prostate revealed upregulation of CD8+ T cells, PD-1/PD-L1 expression, and immune activation. In general terms, MGA271 showed an acceptable safety profile, promising immune-stimulating activity and crosstalk with other immune checkpoints [183]. Results from a phase I/II trial analyzing MGA271 in combination with PD-1-targeted therapy in patients with advanced solid tumors have been published (NCT02475213). One hundred and sixteen of 133 patients experienced treatment-related adverse events and 38 patients were ≥ grade 3. The efficacy of the combination therapy was limited, with an objective response observed in 6 of 18 patients with HNSCC and in 5 of 14 patients with NSCLC who did not receive previous ICI treatment. Only 1 of 17 patients with urothelial cancer and 1 of 13 patients with melanoma showed an objective response, and patients with previous ICI treatment had a poorer prognosis [127]. Unfortunately, a phase II trial analyzing the combination of MGA271 with anti-PD-1 antibody or PD-1xLAG3 bispecific antibody in head and neck cancer (NCT04634825) has just been closed due to seven observed fatalities associated with hemorrhagic events. A more comprehensive evaluation of MGA271 is ongoing in multiple trials, and the further outcome is worth expecting.

Although DS-5573a showed potent ADCC activity in the breast adenocarcinoma xenograft model [184], the only clinical trial evaluating it has been terminated without any released results due to business decisions (NCT02192567). Omburtamab (8H9) is a murine IgG1 monoclonal antibody which was identified to bind 4IgB7-H3 [185]. A humanized version of omburtamab was found to bind to the FG loop of B7-H3 and exhibit potent ADCC activity in neuroblastoma cells when co-cultured with peripheral blood mononuclear cells [186]. Further clinical evaluation of omburtamab’s ADCC activity is not available now, but omburtamab has been the most widely used carrier for radioimmunoconjugates which will be discussed later.

### Targeting B7-H3 with a bispecific antibody

Bispecific antibodies can recognize two different antigens simultaneously to induce synergistic and emergent antitumor activity via various mechanisms including targeting the receptors or engaging immune cells [187]. The structure of bispecific antibodies can be summarized as two or more antibody fragments held together by a linker with or without Fc domains for the fragments to attach to [188]. Multiple forms of B7-H3 targeting bispecific antibodies have been developed preclinically, including CD3/B7-H3 bispecific T cell engagers [189], CD16/B7-H3 bispecific killer cell engager [126], PD-1/B7-H3 bispecific antibody [190] and 4-1BB/B7-H3 bispecific antibody [191]. All these four kinds of agents showed antitumor capacities against tumor cell lines in vitro, while CD3/B7-H3, CD16/B7-H3 and 4-1BB/B7-H3 suppressed tumors in murine xenograft models. A B7-H3 Tri-Specific antibody containing an anti-CD16 fragment, an IL-15 moiety and an anti-B7-H3 scFv has also been developed by Valerra et al. and showed antitumor effects against various tumors in vitro and in a xenograft model [192]. MGD009 is a bispecific T cell engager which simultaneously targets CD3 on T cells and B7-H3. It is currently the only B7-H3 targeting bispecific antibody under clinical evaluation, investigating its synergistic effect with anti-PD-1 therapy in NCT03406949 with no results released yet.

### Targeting B7-H3 via CAR-T therapy

Chimeric antigen receptor T cell (CAR-T) therapy utilizes T cells that have been redirected against the tumor antigen after the engineered expression of CARs to eradicate tumors [193]. CAR-T therapy targeting B7-H3 has shown great potential in a series of studies in preclinical models of multiple cancer types [161, 163, 168, 194, 195], accompanied by a boom in clinical trials confirming the efficacy of B7-H3-targeting CAR-T therapy. Case reports of patients with recurrent anaplastic meningioma, glioblastoma and relapsed basal cell carcinoma who were treated with B7-H3-targeted CAR-T cells have revealed good tolerance and reduced tumor growth [196–198]. A phase I, open-label clinical trial evaluating B7-H3-specific CAR-T cells has just released its early result in ASCO meeting [199]. Sixteen patients with relapsed or refractory non-CNS tumors have been enrolled in two groups and received 0.5 × 10^6^ CAR-T/kg or 1 × 10^6^ CAR-T cells/kg. No dose-limiting toxicity was observed in the first infusion, and maximum circulating CAR-T expansion on the first infusion was 4.98 cells/uL with median persistence of 28 days. Stable disease was observed in 3 of the 9 infused subjects. One subject experienced dramatical CAR-T expansion and transient grade 4 liver enzyme elevation after the second infusion and partial metabolomic response on FDG-PET was observed 28 days later. The biological effective dose has been determined as 1 × 10^6^ CAR-T cells/kg in this trial; still the comparison between two arms needs further enrollment.

### Targeting B7-H3 via radioimmunotherapy

Radioimmunotherapy labels tumor-targeting antibodies with radionucleotides and inhibits tumors through radiation-induced cytotoxicity [200]. Omburtamab is the most frequently used carrier in radioimmunoconjugates as aforementioned. Radioactive iodine labeled Omburtamab has been developed and evaluated in a human rhabdomyosarcoma xenograft model in 2005 [201], where ^131^I-Omburtamab showed specific binding with tumor cell lines and antitumor effects in rhabdomyosarcoma xenograft. Currently, ^131^I-Omburtamab and ^124^I-Omburtamab are the only radioimmunotherapy agents with available clinical evaluation results. In a phase I trial evaluating intrathecal administration of ^131^I-Omburtamab in recurrent metastatic CNS neuroblastoma (NCT00089245), Kramer et al. found that among 80 patients receiving ^131^I-Omburtamab in combination with conventional therapy, 45 (56%) patients remained alive when data were last updated, 45% of patients survive more than 36 months and 29% more than 60 months. The survival data are promising as historical median overall survival time of neuroblastoma in the same institution is 6.6 months. The adverse events in the trial appeared to be manageable, self-limited fever, nausea, headache and transient serum transaminase elevation were observed [202, 203]. Retrospective analysis of the same cohort revealed that intraventricular administration of ^131^I-Omburtamab did not increase the risk of radionecrosis, further confirming the safety of the intervention [204]. In the same institution, a retrospective review of 23 recurrent rhabdomyosarcoma patients also showed prolonged survival of patients receiving intraventricular ^131^I-Omburtamab [205]. The implementation of ^131^I-Omburtamab has also been evaluated in peritoneal tumors (NCT01099644), where intraperitoneal administration of ^131^I-Omburtamab was well tolerated [206]. ^124^I-Omburtamab is another developed radioimmunotherapy agent whose safety and effect in diffuse intrinsic pontine gliomas (DIPG) has been evaluated in phase I clinical trial (NCT01502917). Among the 46 DIPG patients enrolled and treated, 10 patients experienced grade 3 adverse effects which were mainly nervous system disorder. The median overall survival across the cohort was 14.8 months, about 3–4 months longer than historical control data from other trials [207, 208]. Other B7-H3-targeting radioimmunotherapy agents, including ^212^Pb-376.96 [209] and ^131^I-4H7 [210], have also demonstrated promising potential in preclinical models but clinical evaluation is lacking. Further evaluation of ^131^I-Omburtamab and ^177^Lu-DTPA-omburtamab is ongoing, with results yet to be released. Currently, radioimmunotherapy agents targeting B7-H3 were mainly analyzed in CNS and peritoneal tumors, possibly because compartmental administration to reduce systematic exposure is feasible in these tumor types. The management of radio-toxicity remains a great hurdle to overcome when trying to adopt B7-H3 targeting radioimmunotherapy in other solid tumors.

Although all of these approaches are supported by preclinical models, preliminary results of clinical safety and efficacy are available only for ADCC-based MGA271, B7-H3 CAR-T and some radionucleotide-bound antibodies. The number of trials evaluating B7-H3-targeted therapy has increased in recent years, and evidence is accumulating until a comprehensive comparison between different approaches can be made. Substantially supported by promising preclinical results from various cancer models, CAR-T-based B7-H3-targeted therapy is currently the most extensively investigated approach, with 17 phase I/II trials confirming its safety and efficacy. The results from these trials, which might provide novel alternatives and clinical benefits for patients with cancer, are expected.

## B7-H3 in tumor imaging

In addition to serving as a prognostic marker and immunotherapy target, as mentioned above, B7-H3 has also shown clinical application potential in tumor imaging. B7-H3 has been validated as a molecular ultrasound imaging target in breast cancer. In mammography, molecular imaging with ultrasound contrast agents can provide accurate and sensitive imaging signals noninvasively, where microbubbles functionalized with B7-H3-targeted affibody [138] or B7-H3-targeted antibody [211] demonstrated great potential as molecular-targeting contrast agents. In hB7-H3-expressing tumors, microbubbles conjugated to the B7-H3-targeted affibody (MBABY-B7-H3) produced higher imaging signals than nontargeted microbubbles, while in normal mammary tissues and B7-H3-blocking tumors, MBABY-B7-H3 revealed a significantly reduced signal [138], validating the diagnostic value of B7-H3 in breast cancer imaging. With a similar approach, B7-H3-targeted ultrasound imaging was found to be capable of distinguishing metastatic sentinel lymph nodes from nonmetastatic sentinel lymph nodes in a murine breast cancer model [212], which further confirms the potential to evaluate the tumor burden of B7-H3 and indicates imaging value beyond breast cancer of B7-H3. Spectroscopic photoacoustic imaging is another targeted approach that provides sensitive imaging signals based on thermoelastic expansion after laser absorption and subsequent ultrasonic wave emission [213]. By conjugating B7-H3-targeted antibody or affibody to indocyanine green (ICG), a photoacoustic and fluorescence agent, researchers are now able to detect breast cancer [214], evaluate tumor grade [215] and even guide intraoperative resection [216]. Zirconium-89 (89Zr)-labeled anti-B7-H3 monoclonal antibody DS-5573a is another validated B7-H3 targeting imaging approach, where PET/MRI evaluation demonstrated promising in vivo biodistribution and stability of 89Zr-DS-5573a and revealed specific and prolonged targeting of B7-H3-positive tumors [217], further demonstrating the potential of B7-H3 in either target imaging or target immunotherapy, although mainly in the field of breast cancer, B7-H3 has shown its potential in tumor imaging.

## Conclusions

The nature of the B7-H3 receptor remains unknown, which hinders the comprehensive understanding of the role of B7-H3 in the TME and the development of B7-H3-based immunotherapy, warranting further efforts to elucidate the biological characteristics. Nevertheless, the multifaceted role of B7-H3 in the TME has been extensively explored, and B7-H3 has been found to induce malignant behaviors and promote tumor progression through complicated pathways. The role of B7-H3 has been evaluated in tumor cells, T cells, DCs, NK cells, CAFs, neutrophils and endothelial cells in the TME, indicating that B7-H3 is a vital modulator in the TME and a valuable immunotherapy target. Extensive expression of B7-H3 has been reported in a variety of cancer types, and correlation with poor prognosis is also widely established, with the notion that the expression of B7-H3 is heterogenous and that B7-H3 in B7-H3 low-expressing or metastatic cancer needs additional investigation. As dozens of preclinical studies and early-stage trials are ongoing, B7-H3 applications in breast ultrasound imaging and as a serum marker for diagnosis and prognosis prediction have also been identified. Therefore, targeting B7-H3 might provide a novel and promising option for cancer therapy.

## Data Availability

All data and materials used are available from the corresponding author upon reasonable request.

## Abbreviations

5-FU: 5-Fluorouracil
89Zr-: Zirconium-89
ADC: Antibody‒drug conjugates
ADCC: Antibody-dependent cellular cytotoxicity
PKB, Akt: Protein kinase B
ALDH: Aldehyde dehydrogenase
AML: Acute myeloid leukemia
AP-1: Activating protein 1
AR: Androgen receptor
ATP: Adenosine triphosphate
ATRT: Atypical teratoid/rhabdoid tumor
Bax: BCL2-associated X
DTIC: Dacarbazine
Bcl-2: B cell lymphoma-2
Bcl-xl: B cell lymphoma-xl
BiKE: Bispecific killer cell engager
Bmi1: B lymphoma Mo-MLV insertion region 1
CAF: Cancer-associated fibroblast
CAR: Chimeric antigen receptor
CCK-8: Cell counting kit-8
CCL2: CC-chemokine ligand 2
CCR2: CC-chemokine receptor 2
CCRCC: Clear cell renal cell carcinoma
CDC25A: Cell division cycle 25A
CDK4: Cyclin-dependent kinase 4
cDNA: Complementary DNA
CRC: Colorectal cancer
CSCs: Cancer stem cells
CTLA-4: Cytotoxic T lymphocyte antigen 4
CXCL12: CXC motif chemokine 12
DIPG: Diffuse intrinsic pontine glioma
PSA: Prostate-specific antigen
DC: Dendritic cells
DOX: Doxorubicin
ECM: Extracellular matrix
EMT: Epithelial–mesenchymal transition
ENO1: Enolase 1
ERG: E-twenty-six related gene
ERK: Extracellular regulated protein kinases
FASN: Fatty acid synthase
FGF1: Fibroblast growth factor 1
FOXP3: Forkhead transcription factor protein 3
GBM: Glioblastoma
GlUT1: Glucose transporter 1
GM-CSF: Granulocyte-macrophage colony-stimulating factor
GZMB: Granzyme B
HCC: Hepatocellular carcinoma
HHLA2: HERV-H LTR-associated protein 2
HIF-1α: Hypoxia-inducible factor-1
HK2: Hexokinase 2
HNSCC: Head and neck squamous cell carcinoma
HUVEC: Human umbilical vein endothelial cell
ICG: Indocyanine green
ICI: Immune checkpoint inhibitor
IL: Interleukin
IFN: Interferon
IL20RA: Interleukin-20 receptor subunit α
IL20RB: Interleukin-20 receptor subunit β
JAK2: Janus kinase 2
KIF15: Kinesin family member 15
LDHA: Lactate dehydrogenase A
LEPC: Late endothelial progenitor cell
LncRNA: Long noncoding RNA
L-OHP: Oxaliplatin
m6A: N6-methylation of adenosine
MAPK: Mitogen‐activated protein kinase
MB: Medulloblastoma
MCC: Merkel cell carcinoma
MDSC: Myeloid-derived suppressor cell
MEK: Mitogen-activated protein kinase
MHC: Major histocompatibility complex
MMP: Matrix metalloproteinase
mTOR: Mammalian target of rapamycin
MVP: Major vault protein
Neat1: Nuclear-enriched abundant transcript 1
NFAT: Nuclear factor of activated T cells
NF-κB: Nuclear factor-kappa B
NK: Natural killer
Nrf2: Nuclear factor erythroid 2-related factor 2
NSCLC: Non-small cell lung cancer
OS: Overall survival
PD-1: Programmed cell death protein 1
PD-L1: Programmed cell death ligand 1
PD-L2: Programmed cell death ligand 2
PFKFB3: 6-Phosphofructo -2-kinase/fructose-2,6-bisphosphatase 3
PGK1: Phosphoglycerate kinase 1
PLA2R1: Phospholipase A2 receptor 1
PI3K: Phosphatidylinositol 3 kinase
PRX3: Peroxiredoxin 3
sB7-H3: Soluble B7-H3
SIRT1: Sirtuin 1
SOD1: Superoxide dismutase 1
SOD2: Superoxide dismutase 2
SREBP-1: Sterol response element binding proteins-1
SSCs: Spermatogonial stem cells
STAT3: Signal transducer and activator of transcription 3
TAM: Tumor-associated macrophages
TCGA: The Cancer Genome Atlas
TILs: Tumor-infiltrating lymphocytes
TIMP: Tissue inhibitor of metalloproteinase 1
TLT-2, TREML2: Triggering receptor expressed on myeloid cells-like transcript 2
TM4SM1: Transmembrane 4 superfamily member 1
TME: Tumor microenvironment
TNBC: Triple-negative breast cancer
TNF-α: Tumor necrosis factor-alpha
Treg cells: Regulatory T cells
VEGF: Vascular endothelial growth factor
VEGF-A: Vascular endothelial growth factor A
VEGF-D: Vascular endothelial growth factor D
VEGFR2: Vascular endothelial growth factor receptor 2
α-SMA: Alpha-smooth muscle actin
